# Plasma membrane-localized SlSWEET7a and SlSWEET14 regulate sugar transport and storage in tomato fruits

**DOI:** 10.1038/s41438-021-00624-w

**Published:** 2021-08-01

**Authors:** Xinsheng Zhang, Chaoyang Feng, Manning Wang, Tianlai Li, Xin Liu, Jing Jiang

**Affiliations:** 1 College of Horticulture, Shenyang Agricultural University, 110866 Shenyang, Liaoning China; 2 Key Laboratory of Protected Horticulture of Education Ministry, 110866 Shenyang, Liaoning China

**Keywords:** Plant molecular biology, Molecular biology, Transgenic plants, RNAi

## Abstract

Sugars, especially glucose and fructose, contribute to the taste and quality of tomato fruits. These compounds are translocated from the leaves to the fruits and then unloaded into the fruits by various sugar transporters at the plasma membrane. SWEETs, are sugar transporters that regulate sugar efflux independently of energy or pH. To date, the role of SWEETs in tomato has received very little attention. In this study, we performed functional analysis of *SlSWEET7a* and *SlSWEET14* to gain insight into the regulation of sugar transport and storage in tomato fruits. *SlSWEET7a* and *SlSWEET14* were mainly expressed in peduncles, vascular bundles, and seeds. Both SlSWEET7a and SlSWEET14 are plasma membrane-localized proteins that transport fructose, glucose, and sucrose. Apart from the resulting increase in mature fruit sugar content, silencing *SlSWEET7a* or *SlSWEET14* resulted in taller plants and larger fruits (in *SlSWEET7a*-silenced lines). We also found that invertase activity and gene expression of some *SlSWEET* members increased, which was consistent with the increased availability of sucrose and hexose in the fruits. Overall, our results demonstrate that suppressing *SlSWEET7a* and *SlSWEET14* could be a potential strategy for enhancing the sugar content of tomato fruits.

## Introduction

In higher plants, photosynthesis is the process by which carbon and energy and transformed into sugars in source tissues. These sugars are exported to various sink tissues and organs to support normal growth and development^1^. Sugars are an important nutritional component, especially in fruit crop species, and the accumulation of soluble sugars somewhat determines the quality of fruits. However, the accumulation of soluble sugars requires sugar biosynthesis, metabolism, and transport, in which specialized sugar transporters play an indispensable role^2–4^.

Tomato is considered an ideal model crop species because of its fleshy fruits that have important economic value^5^. Sugar transport and storage are essential for improving tomato quality^6,7^. Early studies indicated that sucrose unloading in young tomato pericarps undergoing cell division occurs symplastically^8^. The postphloem cellular pathways in the outer pericarps are associated with switching from the symplastic route during the starch accumulation stage to the apoplastic route during the hexose accumulation stage^9^. This symplastic-to-apoplastic switch is compatible with a facilitated transport process allowing the accumulation of soluble sugars at high concentrations without attenuating phloem unloading^9^.

Sucrose is unloaded from the phloem to the fruit apoplast, where it can be either directly transported into storage parenchyma cells by sucrose transporters (SUTs) through the extracellular pathway or hydrolyzed to glucose and fructose by extracellular invertase and then transported into parenchyma cells by hexose transporters^10,11^. Thus, the apoplastic unloading of sugar can facilitate the influx of hexoses across the plasma membranes of storage cells. In contrast, sucrose can accelerate the efflux of sucrose from the phloem sink to the apoplast through sucrose concentration differences^9^. Three SUTs and three hexose transporters have been identified in tomato^12–14^. Immunolocalization assays showed that three of these SUTs colocalized in sieve elements and that LeSUT1 and LeSUT2 are present only in the sieve tube cells of tomato fruits and not in other phloem cells or storage parenchyma cells^12,15^. However, three hexose transporters (SlHT1, SlHT2, and SlHT3) are expressed in the storage parenchyma cells of tomato fruits^16^. The cleavage of sucrose by sucrose synthase can save more energy than can cleavage by invertase in growing sink organs^17^. Therefore, sucrose transmembrane transport may also occur in the parenchyma cells of tomato fruits.

In tomato, the SUTs and monosaccharide transporters identified thus far are energy dependent and sensitive to the activity of proton transporters. These transporters move sucrose across the membrane by coupling with H^+^-ATPase in the cell membrane. Recent studies have shown that the translocation of sucrose is driven by both saturated (possibly via SUTs) and unsaturated phases, but SUTs and other known monosaccharide transporters display low levels of expression in relevant cells despite showing only saturated sucrose transport kinetics^18,19^. This finding indicates that there are other transporters responsible for the transmembrane transport of sugars, independent of H^+^-ATPase. The discovery of SWEETs (Sugars Will Eventually be Exported Transporters) has provided new insights into sugar transport mechanisms^20^. SWEET proteins are sugar transporters that do not require energy and are responsible for transporting monosaccharides and/or disaccharides across membranes following a concentration gradient^21^. Phylogenetic analysis indicated that the SWEET protein family comprises four clades, the members of which have distinct characteristics (clade I and II SWEETs prefer hexoses; clade III members mainly transport sucrose; and clade IV members are involved in the flux of fructose across the tonoplast)^21,22^. The multiplicity of SWEET genes in higher plants also explains their different functions in essential developmental and physiological processes, including growth and flower, pollen, nectar and seed development^22–29^.

Passive facilitation plays an important role in the release of sucrose from sieve tube companion cell complexes and other vascular bundle cells to fruit parenchyma cells. Among the tomato *SlSWEET* genes, only *SlSWEET1a* and *SlSWEET15* have been experimentally researched, and it has been reported that *SlSWEET1a* is involved in sugar regulation in fruits and leaves and that *SlSWEET15* is responsible for fruit and seed development^7,30,31^. Therefore, it is necessary to explore other candidate *SWEET* genes involved in sustaining sugar homeostasis in tomato fruits to improve their quality. In this study, *SlSWEET7a* and *SlSWEET14* were selected for further analysis based on the results of a preliminary study^32^. Here, the putative effects of *SlSWEET7a* and *SlSWEET14* on tomato fruits were explored.

## Results

### Expression analysis of *SlSWEET7a* and *SlSWEET14* during fruit development

**Fig. 1:**
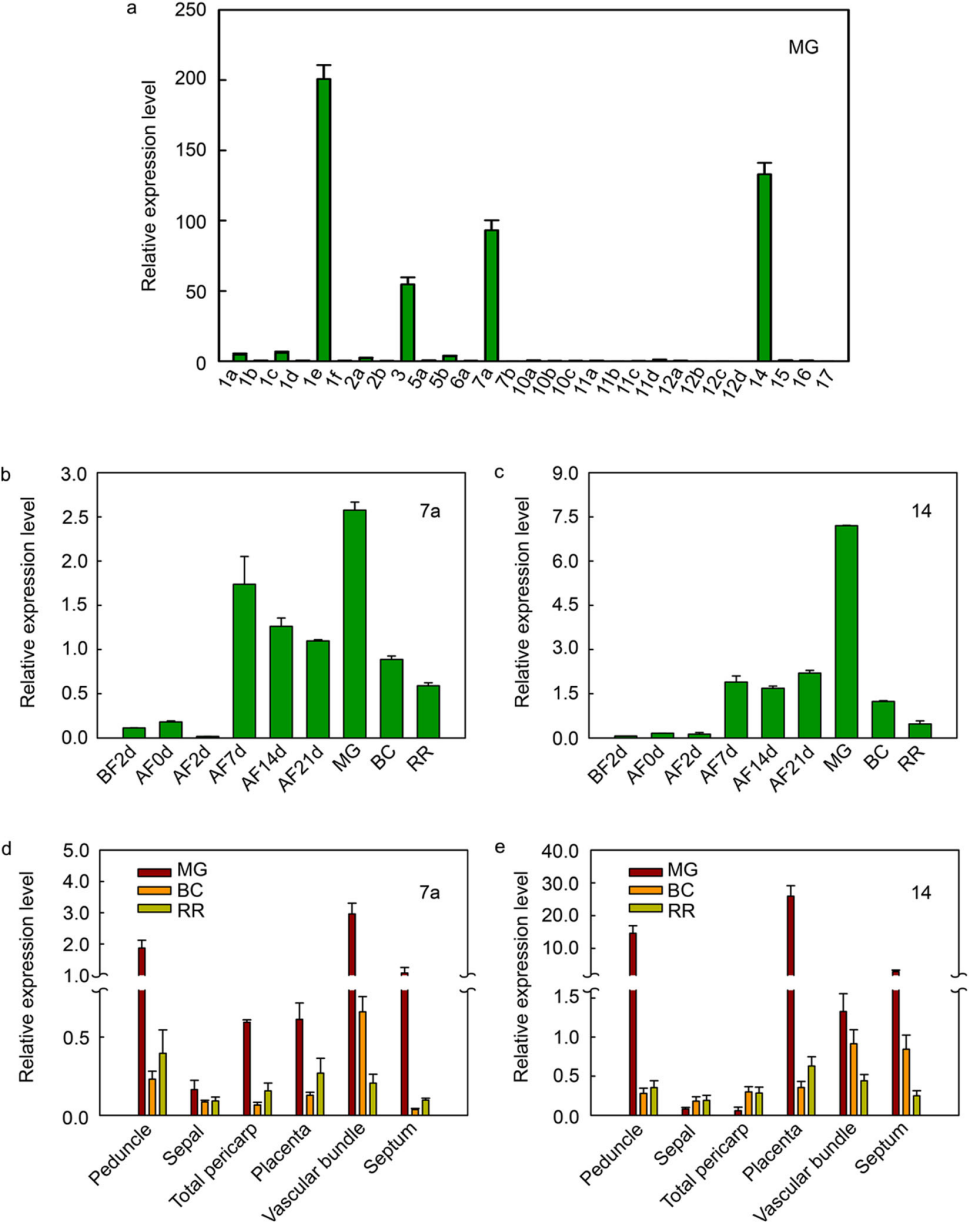
Expression pattern analysis of *SlSWEET* genes. **a** At the transcript level, *SlSWEET* genes were found to exist as 29 different members in Micro-Tom mature green (MG) fruits. **b**, **c** Relative expression of *SlSWEET7a* and *SlSWEET14* during fruit development in Micro-Tom. BC2d, 2 days before flowering; AF0d flowering, AF2d 2 days after flowering, AF7d 7 days after flowering, AF14d 14 days after flowering, AF21d 21 days after flowering, MG mature green, BC breaking color, RR red ripe. **d**, **e** Gene expression of *SlSWEET7a* and *SlSWEET14* in different tissues during fruit development in Micro-Tom. Different tissues such as the peduncles, sepals, total pericarp (including pulp and pericarp), placentas, vascular bundles, and septa were investigated during the MG, BC, and RR stages. The data are shown as the means ± SDs of three independently biological replicates

The results of quantitative real-time PCR analysis of 29 *SlSWEETs* in MG Micro-Tom fruits (Fig. 1a) showed that *SlSWEET1e*, *SlSWEET3*, *SlSWEET7a*, and *SlSWEET14* transcripts were more abundant than those of the other *SlSWEETs*. SlSWEET1e and SlSWEET3 belong to clade I, whose members are involved in glucose import^7,30,32^, while SlSWEET7a and SlSWEET14 belong to clade II and clade III, respectively; the functions of these two clades have not yet been identified in tomato. Therefore, we selected *SlSWEET7a* and *SlSWEET14* as genes of interest for further study.

To further explore the effects of these two genes on the development of tomato fruits, we performed an expression pattern analysis of different fruit tissues at different fruit developmental stages and (Fig. 1b, c). *SlSWEET7a* and *SlSWEET14* showed similar expression patterns during fruit development. Seven days after flowering, the expression levels of both genes first peaked and then peaked again in the MG stage. However, their transcript levels decreased after the MG stage. These results suggest that these two genes might play a role during early fruit development and carbohydrate accumulation.

Subsequently, we analyzed the expression of the same two genes in different tomato fruit tissues (Fig. 1d, e). The transcript level of *SlSWEET7a* was higher in all tissues at the MG stage than in those at the BC or RR stages and was relatively high in the peduncles and septa, especially in the vascular bundles, compared with the other tissues at the MG stage. *SlSWEET14* had a similar expression pattern. The transcript level of *SlSWEET14* was markedly higher in the peduncles, vascular bundles, septa, and, especially, placentas, at the MG stage than in other tissues and at other stages. These results indicate that *SlSWEET7a* and *SlSWEET14* could play important roles in sugar accumulation from the mature to ripe stages.

**Fig. 2:**
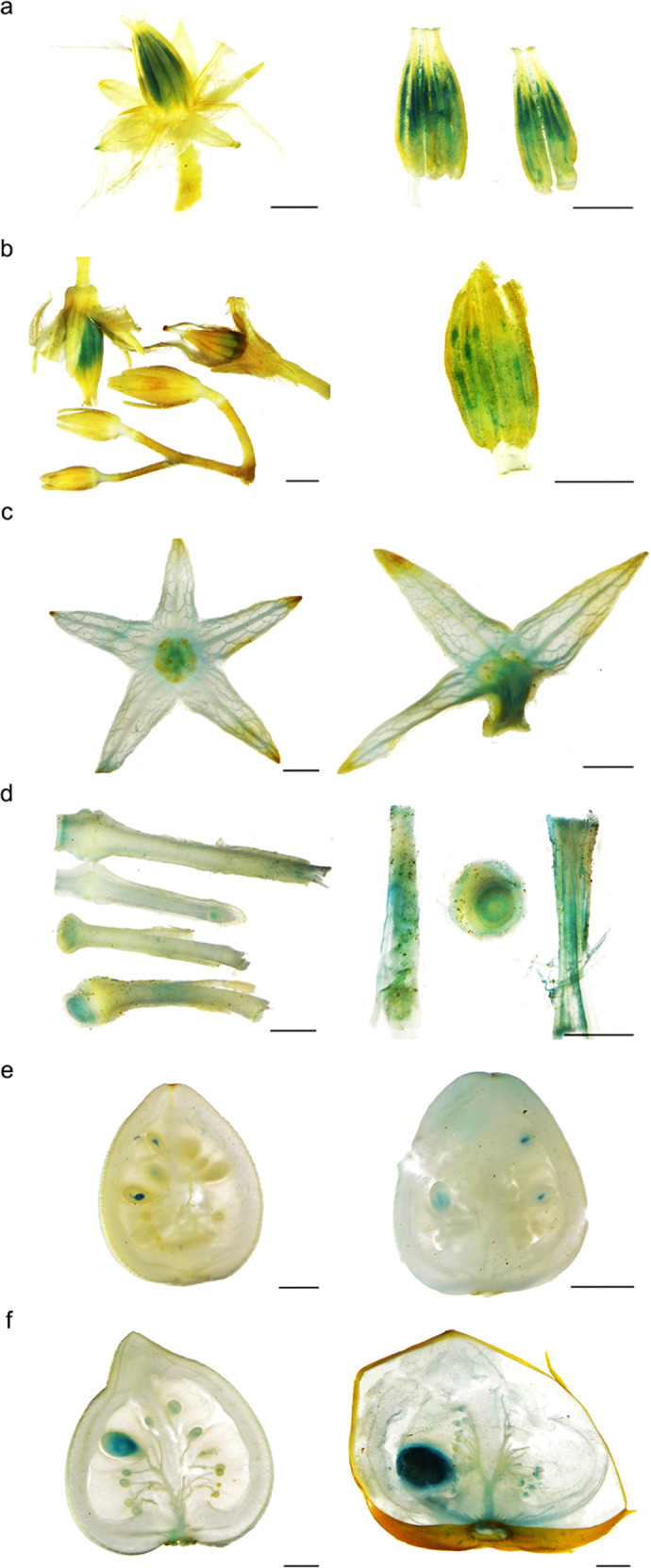
Histochemical analysis of GUS activity in transgenic tomato plants. **a** Histochemical staining of GUS activity driven by the promoter of *SlSWEET7a* in flowers. **b**–**f** Histochemical staining of GUS activity driven by the promoter of *SlSWEET14* in different tissues. **b** Flowers, **c** sepals, **d** peduncles, and **e** fruits of the expanding period and **f** fruits of the mature green stage (left) and red ripe stage (right). The scale bars correspond to 2000 μm

To investigate the spatial expression patterns of *SlSWEET7a* and *SlSWEET14*, we generated transgenic plants expressing the GUS gene under the control of the *SlSWEET7a* and *SlSWEET14* promoters (*pSlSWEET7a:GUS* and *pSlSWEET14:GUS*, respectively) (Fig. 2). The promoter activities of *SlSWEET7a* and *SlSWEET14* were measured in the stamens during the later stages of flower development. A closer investigation revealed that this expression was localized in the anthers. In *pSlSWEET14:GUS* transgenic fruits, GUS activity was detected around vascular tissues in the peduncles, sepals, and pericarps. GUS staining was also observed in seeds at different stages. A transient GUS assay showed that the expression patterns of GUS genes driven by the promoters of the two genes were nearly identical and were mainly distributed in the vascular bundles and seeds (Supplementary Fig. 1). The expression patterns of *SlSWEET7a* and *SlSWET14* showed that they may be involved in sugar accumulation and seed development.

### Plasma membrane-localized SlSWEET7a and SlSWEET14 proteins

**Fig. 3:**
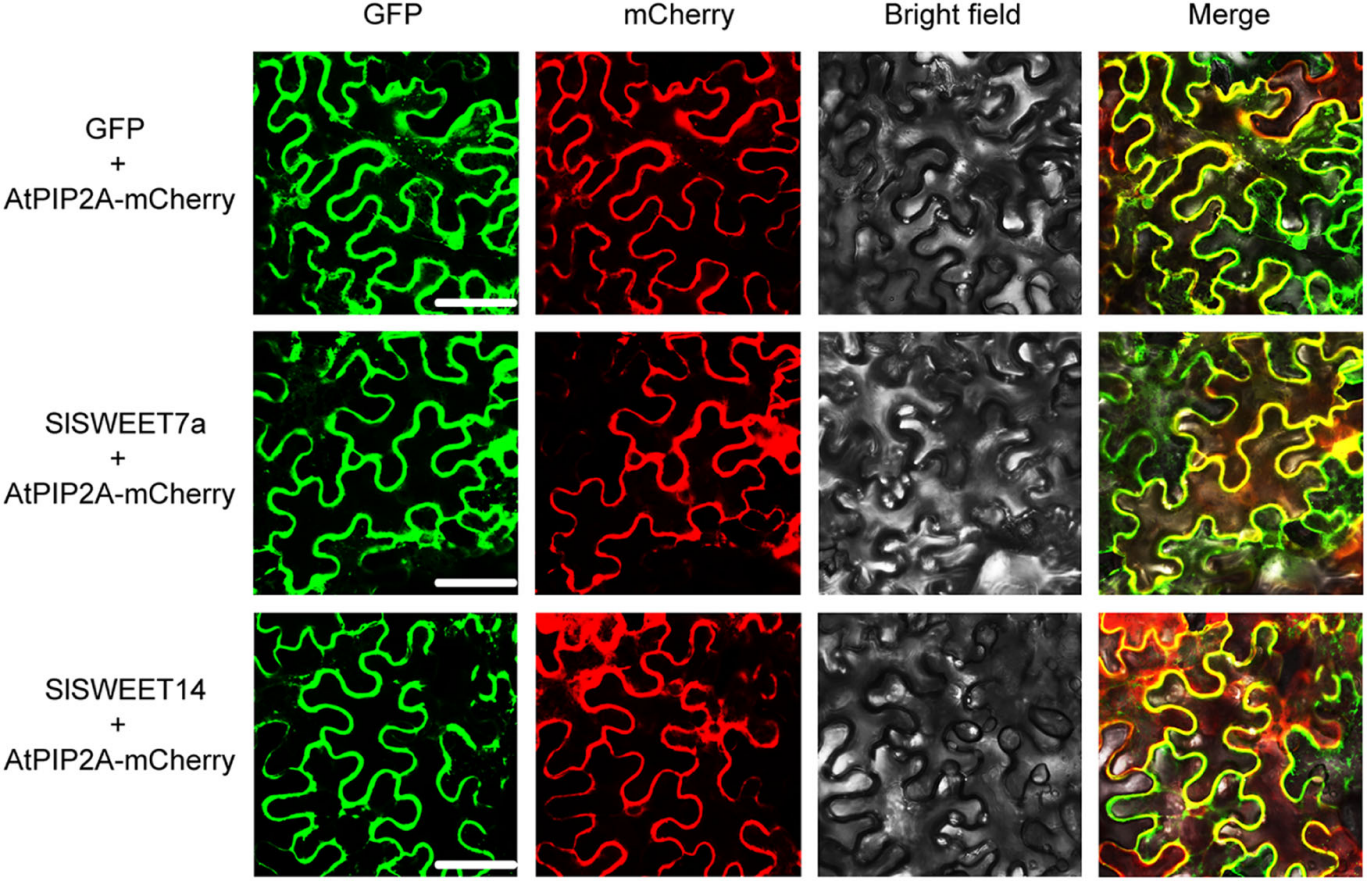
Analysis of the subcellular localization of SlSWEET7a and SlSWEET14. Confocal images of *N. benthamiana* leaves expressing SlWEET7a and SlWEET14. An mCherry-labeled plasma membrane marker (AtPIP2A) was coexpressed to visualize the plasma membrane. An empty vector was used as a positive control. The experiment was performed independently three times, each yielding similar results. The scale bars correspond to 50 μm

To confirm the intracellular localization of SlSWEET7a and SlSWEET14, the subcellular localization of these two SWEETs was determined in *Nicotiana benthamiana* epidermal cells. Coexpressed pCAM35S::SlSWEET7a-GFP or pCAM35S::SlSWEET14-GFP, together with a mCherry-labeled plasma membrane marker protein (pCAM35S::AtPIP2A-mCherry), was introduced into the epidermal cells of *N. benthamiana* leaves. A pattern of green fluorescence consistent with the predominant localization of green fluorescent protein (GFP) was observed in the plasma membrane, while additional minor fluorescence was associated within the cytoplasm. The red fluorescence of PM-mCherry overlapped with the green fluorescence of SlSWEET7a and SlSWEET14-GFP, indicating that SlSWEET7a and SlSWEET14 were mainly localized in the plasma membrane (Fig. 3).

### Transport substrate specificity of SlSWEET7a and SlSWEET14 in yeast

**Fig. 4:**
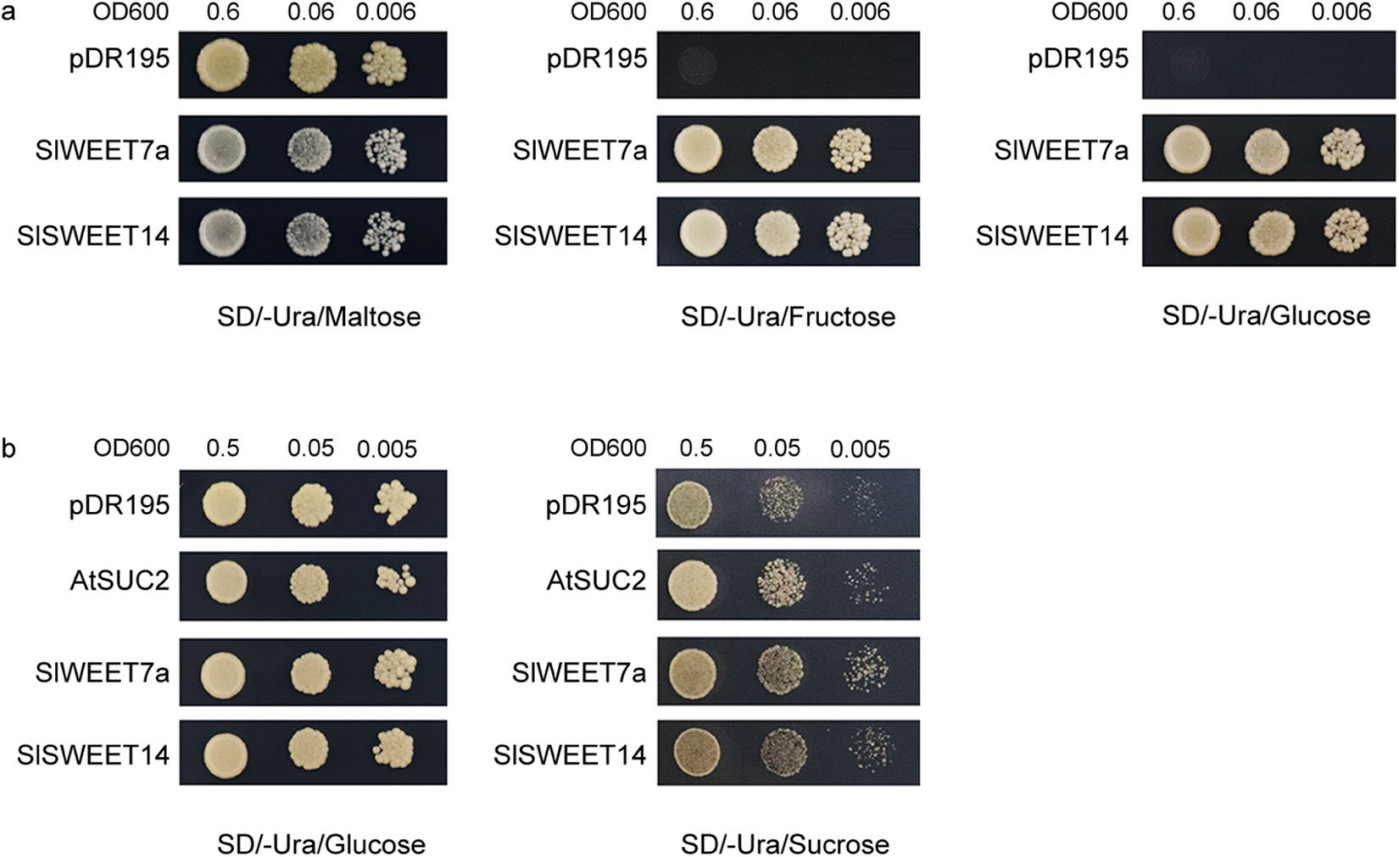
Analysis of the transport activity of SlSWEET7a and SlSWEET14 in yeast cells. **a** Growth of the yeast mutant strain EBY.VW4000 expressing different genes in SD (-Ura) media supplemented with different carbon sources*-*2% maltose, 2% fructose, or 2% glucose; yeast mutant strains transformed with the pDR195 empty vector were used as negative controls. **b** Growth of the yeast mutant strain SUSY7/ura3 expressing different genes in SD (-Ura) media supplemented with 2% glucose or 2% sucrose. An empty vector (pDR195) was used as a negative control, and AtSUC2 was used as a positive control. Yeast cells of strain EBY.VW4000 were grown at 30 °C for 3 days, and yeast cells of strain SUSY7/ura3 were grown at 30 °C for 4 days

The functionality of the two SlSWEETs in taking up hexose and sucrose was examined via heterologous expression of their respective cDNAs in a hexose transport-deficient yeast strain (EBY.VW4000) and in the cells of a mutant SUSY7/ura3 yeast (*Saccharomyces cerevisiae*) strain that is deficient in the wild-type sucrose uptake mechanism (external sucrose as the sole carbon source cannot be utilized since extracellular invertase is lacking) and has sucrose synthase activity to metabolize any sucrose taken up by foreign sucrose transporters. The results showed that the expression of *SlSWEET7a* and *SlSWEET14* restored the growth of EBY.VW4000 on media supplemented with glucose or fructose (Fig. 4a). Compared with their corresponding cells transformed with the control vector, the SUSY7/ura3 cells transformed with pDR195 containing each of the two SlSWEET homologs (SlSWEET7a and SlSWEET14) and AtSUC2 (used as a positive control) exhibited faster growth on media containing sucrose as the sole source of carbon (Fig. 4b). These results indicate that SlSWEET7a and SlSWEET14 can transport hexose and sucrose.

### Identification of potential interactions between SlSWEET7a and SlSWEET14

**Fig. 5:**
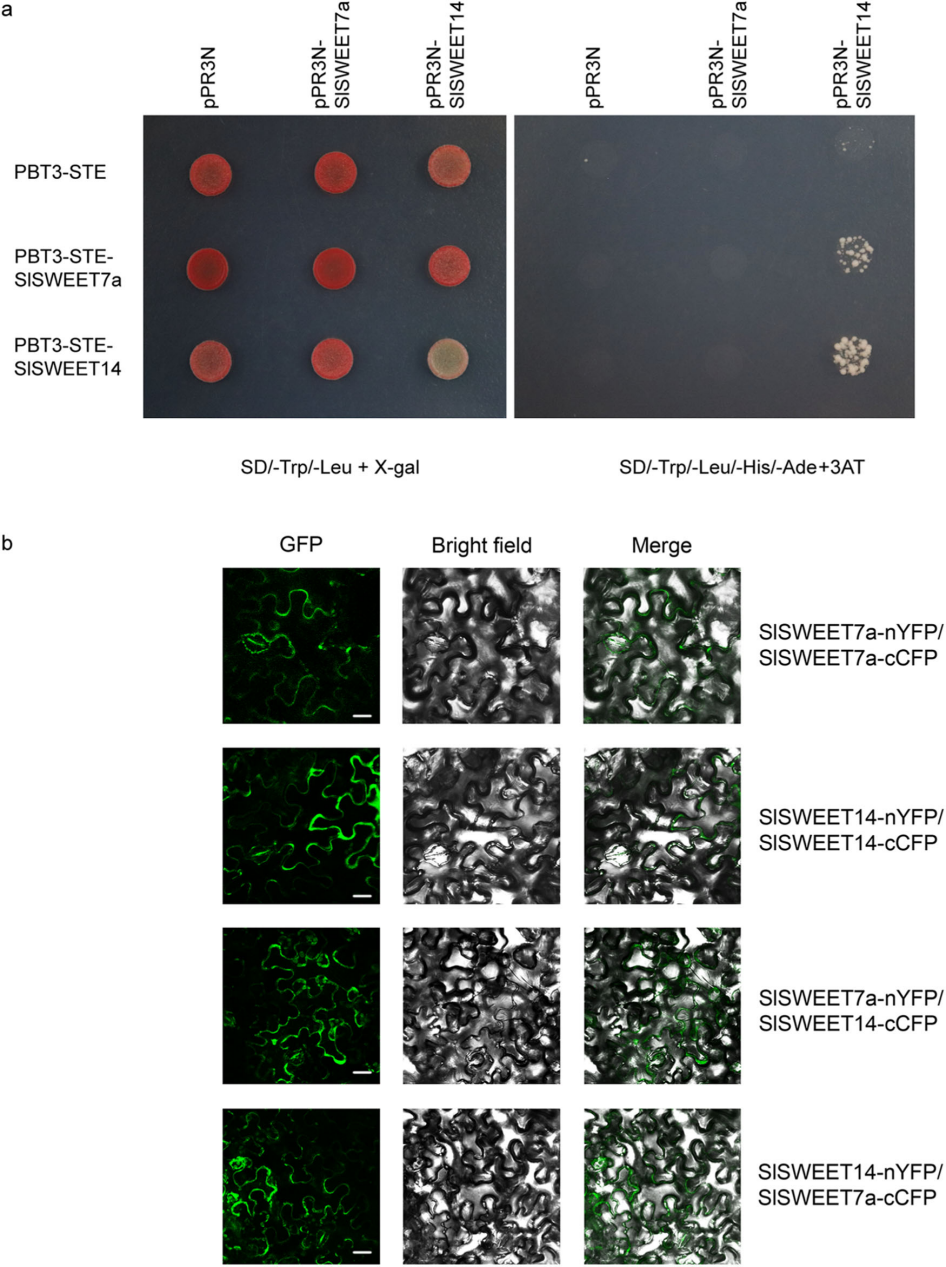
Identification of interactions between the SlSWEET7a and SlSWEET14 proteins. **a** Interaction assays of SlSWEET7a and SlSWEET14 proteins via split-ubiquitin yeast two-hybrid assays. The interactions were tested using His and Ade reporter genes and 3-amino-1,2,4-triazole (10 mM) (right panel) and verified using an X-gal (100 µg/ml) staining assay (left panel). pR3N together with pBT3-STE, pR3N-SlSWEET7a together with pBT3-STE, pR3N-SlSWEET14 together with pBT3-STE, pR3N together with pBT3-SlSWEET7a, and pR3N together with pBT3-SlSWEET14 were used as negative controls. The experiment was performed at least three times, and representative results are shown. **b** Bimolecular fluorescence complementation assays for the interaction of SlSWEET7a and SlSWEET14. Reconstitution of GFP-derived fluorescence and bright field and merged images are shown on the left, middle, and right, respectively. The green signal indicates GFP fluorescence. The scale bars correspond to 25 μm

Given that other known SWEET proteins in *Arabidopsis* compose homo- or heterooligomeric complexes^33^, it is conceivable that SlSWEETs function as higher-order oligomers (e.g., dimers) to perform sugar transport in tomato fruits. To confirm any interactions between the SlSWEET7a and SlSWEET14 proteins, a split-ubiquitin membrane yeast two-hybrid (MYTH) system was used (Fig. 5a). The results indicate that at least SlSWEET14 can form homooligomers.

To determine whether interactions can also occur *in planta*, oligomerization was tested for SlSWEET7a and SlSWEET14 using a split-GFP assay. The NH_2_-proximal half of YFP (nYFP) and C-proximal half of CFP (cCFP) were fused to the C-termini of SlSWEET7a and SlSWEET14, and the fusion proteins were transiently coexpressed in *N. benthamiana* leaves. Homooligomerization of SlSWEET14 was observed in the split-GFP assay. Interestingly, SlSWEET7a was found to form homooligomers and heterooligomers with SlSWEET14 *in planta* (Fig. 5b and Supplementary Fig. 2), whereas no oligomerization was detected in the yeast two-hybrid system. All interactions seemed to occur at the plasma membrane. These split-GFP data also confirm that SlSWEET7a and SlSWEET14 are mainly localized in the plasma membrane.

### Generation of RNAi-mediated suppression lines for *SlSWEET7a* and *SlSWEET14*

**Fig. 6:**
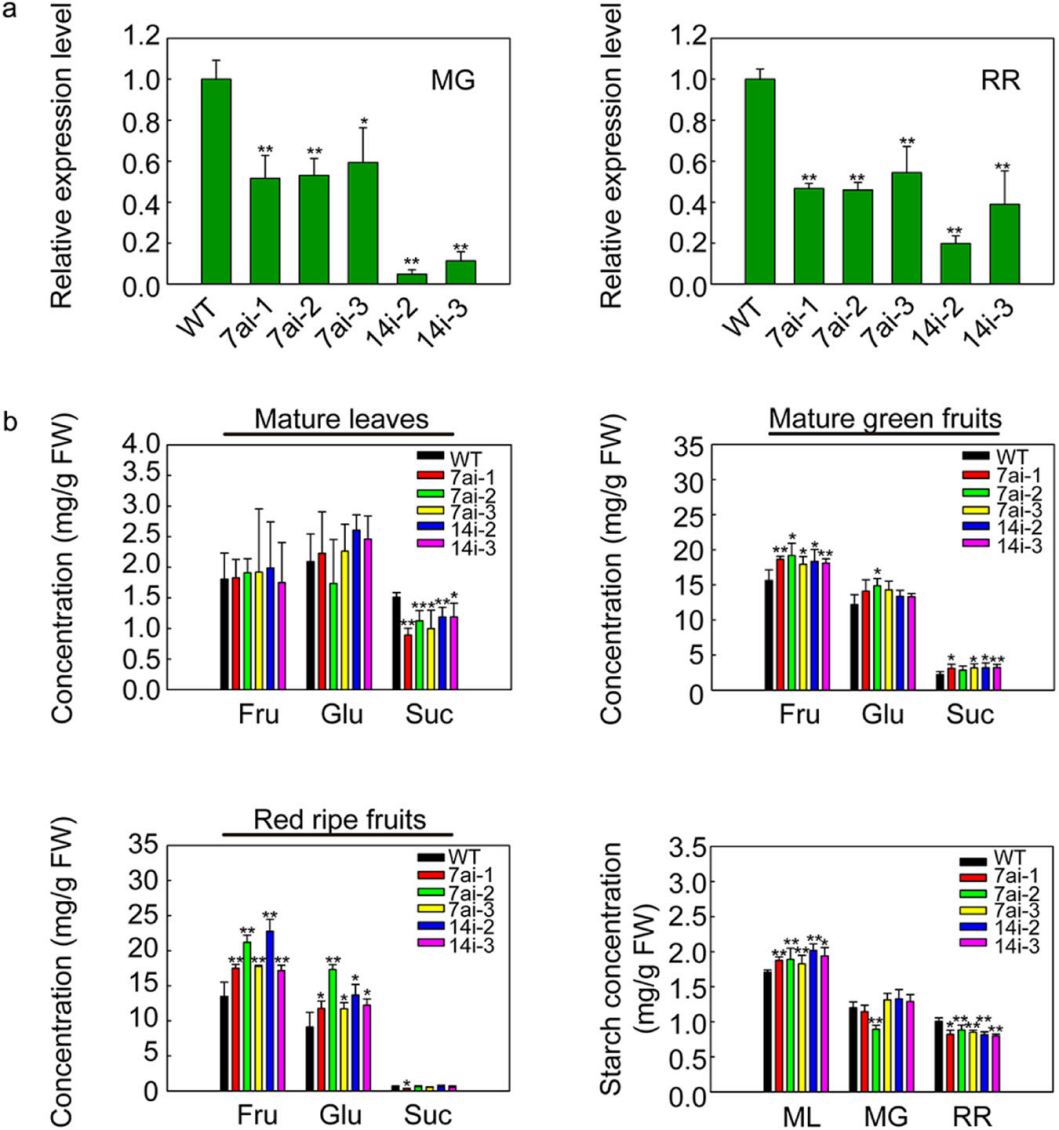
Sugar concentrations in wild-type and RNAi lines. Three independent silenced lines of SlSWEET7ai (SlSWEET7ai-1, SlSWEET7ai-2, and SlSWEET7ai-3) and two lines of SlSWEET14i (SlSWEET14i-2 and SlSWEET14i-3) were selected for experiments. **a** Transcript levels of *SlSWEET7a* and *SlSWEET14* in *SlSWEET7a*- and *SlSWEET14*-silenced fruits at the mature green stage and red ripe stage, respectively. The WT (wild type) expression data were normalized to 1. The *ACTIN* gene was used as the internal control. **b** Soluble sugar concentrations (fructose, glucose, and sucrose) and starch concentrations of the wild-type and RNAi lines in mature leaves (ML), mature green fruits (MG), and red ripe fruits (RR). 7ai, RNAi lines of *SlSWEET7a*; 14i, RNAi lines of *SlSWEET14*. The data represent the means ± SDs of at least five biological replicates. The asterisks indicate *P*-values (*P < 0.05; **P < 0.01) according to Student’s *t* test. FW, fresh weight

To suppress the specificity of *SlSWEET7a* and *SlSWEET14* in tomato plants, two RNAi-mediated vectors were constructed using cDNA sequences (Supplementary Fig. 3). In total, 7 and 15 transgenic lines were generated from the T_1_ generation of *SlSWEET7a*-RNAi and *SlSWEET14*-RNAi, respectively. To further confirm that the transcript levels of these two genes indeed decreased in the MG and RR fruits of the transgenic lines (SlSWEET7ai or SlSWEET14i), we performed quantitative real-time PCR analysis (Fig. 6a). We found that the expression levels of *SlSWEET7a* and *SlSWEET14* significantly decreased in the silenced MG and RR fruits, respectively. Finally, three independent lines with an RNAi construct for *SlSWEET7a* and two for *SlSWEET14* in the T_4_ generation were selected to analyze target gene suppression and for further analysis of phenotypes and sugar concentrations.

### Tomato sugar and starch concentrations and phenotypes were altered

We analyzed the soluble sugar (fructose, glucose, and sucrose) and starch concentrations in the T_4_ generation of the RNAi-silenced *SlSWEET7a* and *SlSWEET14* lines (Fig. 6b). Silencing the genes did not significantly influence the hexose concentration but significantly decreased the sucrose concentration in the mature leaves of all the transgenic lines. The starch concentrations of both SlSWEET7ai and SlSWEET14i were significantly higher in the mature leaves compared with other organs, where the concentrations increased by ~7–10% in SlSWEET7ai and 13%-18% in SlSWEET14i compared with those in the WT.

In the MG fruits of all the silenced lines, significant increases in fructose concentrations and but marked differences in glucose concentrations were detected, except for the 7ai-2 lines. The fructose concentration increased by ~19, 22, and 15% in the three SlSWEET7ai MG fruits compared with the WT (wild type) fruits and by ~17 and 16% in the SlSWEET14i fruits. The sucrose concentration also considerably increased in all the silenced lines compared with the WT, except the 7ai-2 lines, and increased by ~40–44%.

In RR fruit tissues, hexose (fructose and glucose) concentrations considerably increased in all the RNAi lines compared to the WT. The glucose and fructose concentrations increased by ~30 and 60%, respectively, in the SlSWEET7ai RR fruits compared with the WT fruits, and those of the SlSWEET14i fruits increased by ~60 and 30%, respectively. There were no significant differences in sucrose concentration of RR fruits among any of the RNAi lines except for the 7ai-1 line. The starch concentrations of both SlSWEET7ai and SlSWEET14i were significantly lower in the RR fruits than in the WT fruits. The starch concentrations in the RR fruits of the SlSWEET7ai and SlSWEET14i lines decreased by ~12–18% and 18–20%, respectively, relative to those in WT fruits.

**Fig. 7:**
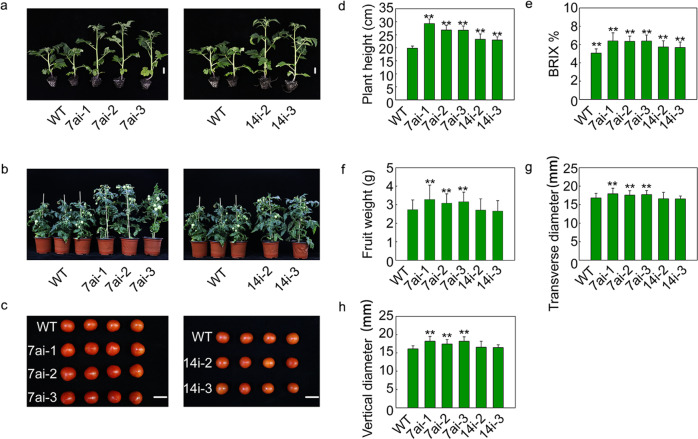
Morphological phenotypes and growth parameters of RNAi lines for silencing *SlSWEET7a* and *SlSWEET14*. Three independent silenced lines of SlSWEET7ai (SlSWEET7ai-1, SlSWEET7ai-2, and SlSWEET7ai-3) and two independent lines of SlSWEET14i (SlSWEET14i-2 and SlSWEET14i-3) were selected for experiments. **a** Plant architecture of the 7ai lines (SlSWEET7ai-1 lines, SlSWEET7ai-2 lines, and SlSWEET7ai-3) and the 14i lines (SlSWEET14i-2 lines and SlSWEET14i-3i lines) at 30 days after sowing the seeds; the scale bars correspond to 2 cm. **b** Plant architecture of SlSWEET7ai lines at 90 days after sowing the seeds. **c** Fruits from nontransgenic and transgenic lines at the red ripe fruit stage; the scale bars correspond to 2 cm. **d** Plant height (*n* ≥ 12) ~90 days after sowing seeds of the SlSWEET7ai lines and SlSWEET14i lines. **e** Soluble solid content (indicated by Brix %) of fruits (*n* ≥ 20) at the red ripening stage. **f** Individual-fruit weight (*n* ≥ 35). **g**, **h** Transverse diameter and vertical diameter of fruits (*n* ≥ 35). The data represent mean values ±SDs. The asterisks indicate *P*-values (*P < 0.05; **P < 0.01) according to Student’s *t* test

During the growth and development of tomato plants, the height of the SlSWEET7ai and SlSWEET14i plants was significantly greater than that of the WT plants from the vegetative growth stage to the reproductive stage (Fig. 7a, b). The height increased by 35–48% in SlSWEET7ai and by 16–17% in SlSWEET14i relative to that of the WT (Fig. 7d). The soluble solid contents (Brix %) also greatly increased in all SlSWEET7ai and SlSWEET14i lines (Fig. 7e). The individual-fruit weight and fruit diameter of the SlSWEET7ai lines were notably greater than those of the WT (Fig. 7f–h). Indeed, the SlSWEET7ai fruits were larger than the WT fruits (Fig. 7c). These results indicate that silencing *SlSWEET7a* and *SlSWEET14* could result in phenotypic differences during both vegetative and reproductive growth stages.

### Effects of silencing SlSWEET7a and SlSWEET14 on genes related to sucrose metabolism or transport

**Fig. 8:**
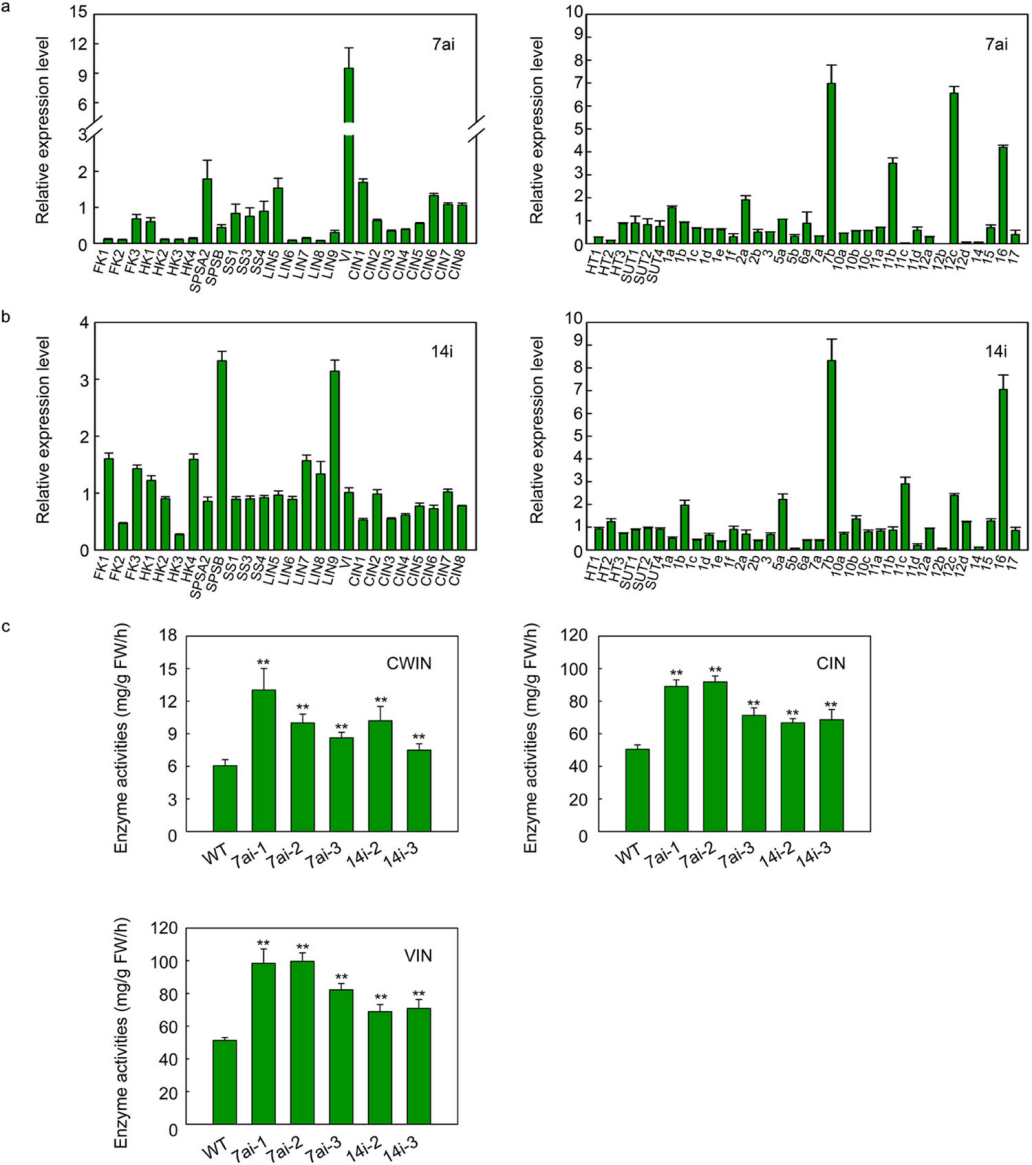
Relative expression levels of genes involved in sucrose metabolism and transport as well as activities of enzymes in mature green fruits. Mixtures of cDNA from three SlSWEET7ai lines or two SlSWET14i lines were used for experiments. **a** Transcript levels of sucrose metabolism (left)- and transport (right)-related genes in SlSWEET7ai (7ai) fruits at the mature green stage. **b** Transcript levels of sucrose metabolism (left)- and transport (right)-related genes in SlSWEET14i (14i) fruits at the mature green stage. FK fructokinase, HK hexokinase, SPS sucrose phosphate synthase, SS sucrose synthase, LIN cell wall invertase, VI vacuolar invertase, CIN cytoplasmic invertase, HT hexose transporter, SUT sucrose transporter, 1a-17 Sugar will eventually be exported transported (SWEET). The expression data of the WT (wild type) were normalized to 1. The *ACTIN* gene was used as an internal control. **c** Enzyme activity assay of mature green fruits from silenced lines. CWIN cell wall invertase, CIN cytoplasmic invertase, VI vacuolar invertase. The data represent the means ± SDs of at least three biological replicates. The asterisks indicate *P*-values (*P < 0.05; **P < 0.01) according to Student’s *t* test

We selected genes related to sugar metabolism and transport and studied their transcription to better understand the changes in sugar levels (Fig. 8a, b). Sucrose metabolism is the process through which sucrose is degraded and resynthesized in the cytosol, vacuole, and apoplast. We analyzed the specific genes related to sugar metabolism, including 14 invertases (LIN5-9, VI, and CIN1-8), three sucrose synthases (SS1, SS2, and SS4), two sucrose phosphate synthases (SPSA2 and SPSB), four hexokinases (HK1-4), and three fructokinase genes (FK1-3). The expression of nearly half of the identified genes (*FK1*, *FK2*, *HK2*, *HK3*, *HK4*, *SPSB*, *LIN6-9*, and *CIN3-5*) was downregulated, while that of several other detected genes (*SPSA2*, *LIN5*, and *VI*) was upregulated in the SlSWEET7ai MG fruits. Among them, *VI* was obviously upregulated by ~8-fold. The expression of these genes showed different patterns in SlSWEET14i MG fruits. The transcript levels of *FK1*, *FK3*, *HK4*, *SPSB*, *LIN7,* and *LIN9* were higher in the SlSWEET14i lines compared with the other lines, while those of *FK2*, *HK3*, *CIN1*, *CIN3*, and *CIN4* were lower. These findings suggest that SlSWEET7a and SlSWEET14 regulate sucrose metabolism by modulating the expression of a specific set of genes associated with sucrose degradation and synthesis.

In addition, sugar transporters play an indispensable role in the accumulation of soluble sugars. SlHT1-3 are the main contributors to hexose accumulation in tomato fruits^16^. The transcript levels of *SlHT1* and *SlHT2* were obviously downregulated in SlSWEET7ai tomato MG fruits. The expression of three sucrose transporter-encoding genes (*SlSUT1*, *SlSUT2*, and *SlSUT4*) was also analyzed. Among them, there were no significant changes in the expression of the three genes encoding sucrose transporters in MG fruits of SlSWEET7ai or SlSWEET14i tomato. The expression of all *SWEET* genes was analyzed in the RNAi lines. Of the clade I members encoding transporters that facilitate glucose transport, *SlSWEET1a* and *2a* were highly expressed, while others were predominantly downregulated in the MG fruits of the SlSWEET7ai lines. Of the potential *SlSWEET* genes encoding sucrose transporters from clade III, *SlSWEET11b* and *SlSWEET12c* also significantly increased. The expression levels of *SlSWEET7b* and *SlSWEET16*, putative hexose-transporting SWEETs belonging to clades II and VI, respectively, were also high in the SlSWEET7ai line. Among the *SlSWEET* genes investigated, the transcript levels of *SlSWEET1b*, *5a*, *7b*, *11c*, *12c*, and *16* genes significantly increased in the SlSWEET14-RNAi lines. These findings suggest that the silencing of *SlSWEET7a* or *SlSWEET14* also influences the expression of other members of the SWEET family.

### Effects of silencing SlSWEET7a and SlSWEET14 on invertase activity

To better understand why the hexose concentration changed in the *SlSWEET7a* and *SlSWEET14* RNAi lines, invertase enzyme activities were analyzed. As shown in Fig. 8c, the activity of three invertase enzymes (cell wall invertase, cytoplasmic invertase, and vacuolar invertase) significantly increased in comparison to that in the WT. Cell wall invertase activity increased by 42–115% in the SlSWEET7ai line and by 23–68% in the SlSWEET14i line; cytoplasmic invertase activity increased by 41–82% in the SlSWEET7ai lines and 32–35% in the SlSWEET14i line; and vacuolar invertase activity increased by 60–94% in the SlSWEET7ai line and 34–38% in the SlSWEET14i line. These results indicated that silencing *SlSWEET7a* and *SlSWEET14* could improve invertase activity and promote sucrose cleavage in the intracellular and extracellular space.

## Discussion

### Plasma membrane-localized SWEET7a and SlSWEET14 in tomato could transport hexoses and sucrose

Previous studies have shown that most SWEET proteins are localized on the plasma membrane and may be the primary site at which sugar flux is regulated^7,30^. SWEET proteins, which have been identified as bidirectional transporters, can regulate both cellular uptake and efflux of sugars, but there are differences in substrate specificity between the different SWEETs^22,34^. Phylogenetic analysis has indicated that SlSWEET7a and SlSWEET14 belong to clades II and III, respectively^30^. Our results showed that these two SWEETs are located on the plasma membrane and are capable of transporting hexose (glucose and fructose) and sucrose, similarly to clade III members AtSWEET11 and AtSWEET12^35^. In addition, VvSWEET10 from clade III is a hexose-affinity transporter in grape^36^. Accordingly, the substrate specificity of SWEETs may also be dependent on species, not merely on SWEET clade. SWEET proteins have a broad range of substrate transport functions and can transport not only sugars but also phytohormones (gibberellins) and other substrates^3,37^.

SWEETs assemble into oligomers to establish translocation pores to transport sugars, especially for larger substrates such as sucrose or fructose^34,38^. Coimmunoprecipitation experiments have suggested that both OsSWEET2b and AtSWEET1 can form homooligomers^39^. Split-ubiquitin yeast two-hybrid and split-GFP assays have also demonstrated homo- and heterooligomerization of SWEETs in *Arabidopsis thaliana*^33,39^. We detected similar interactions between SlSWEET7a and SlSWEET14. The split-ubiquitin system and split-GFP system showed that SlSWEET14 can form homooligomers. The split-GFP system indicated that SlSWEET7a and SlSWEET14 can form heterooligomers, but the split-ubiquitin system did not. Similarly, AtSWEET6 homooligomerization was observed only in the split-GFP assay, and AtSWEET4 could form homooligomers in a split-GFP system; however, this was not observed in a yeast two-hybrid system^33^. Therefore, whether the oligomerization of SlSWEET7a and SlSWEET14 plays an important role in transporting hexoses across the cell membranes of tomato fruits requires further investigation.

### *SlSWEET7a* and *SlSWEET14* mediate sugar transport and metabolism

There are an increasing number of reports concerning SWEET functions in sink organs, especially fleshy fruits. In *Ananas comosus*, 12 of the 21 *AnmSWEETs* were shown to be expressed in different stages of fruit development and ripening. *AnmSWEET5* and *AnmSWEET11* are especially highly expressed in the early stages of fruit development^40^. In apple, *MdSWEET9b* and *MdSWEET15a* likely regulate fruit sugar accumulation^41^. Overexpressing *VvSWEET10* improved hexose accumulation in grapevine calli and tomato fruits^36^. In tomato, overexpressing plasma membrane-localized SlSWEET1a promoted glucose efflux from fruits, resulting in an altered hexose composition in ripening tomato fruits^7^. Interestingly, hexose accumulation also increased in *SlSWEET7a-* and *SlSWEET14*-silenced tomato fruits in the present study. The physiological roles of the two genes in tomato fruits may also involve hexose efflux. Our findings showed that *SlSWEET7a* and *SlSWEET14* were expressed at the highest levels at the MG stage. *SlSWEET7a* was expressed specifically in the peduncle and vascular bundle tissues, and *SlSWEET14* was expressed specifically in the peduncles and especially in the placenta, vascular bundles, and seeds. In the placental phloem parenchyma of tomato fruits, where sucrose is unloaded via an apoplastic pathway, the phloem parenchyma located on the border between the placenta and the seed is a possible site for the apoplastic unloading of sucrose^42^.

Unloaded sucrose can be either directly transported into fruit parenchyma cells by SUTs or hydrolyzed into fructose and glucose by cell wall invertase (CWIN) in the apoplast and then transported into parenchyma cells by hexose transporters (HTs)^1^. Excessive amounts of hexose inhibit the growth of reproductive tissues such as pollen tubes^43^. The coexpression of *SlHTs* and *SlSWEETs* may be required to sustain cytosolic sugar homeostasis^44^. In this study, we observed that hexose concentrations increased in the MG fruits or RR fruits of the silenced lines. As expected, cell wall invertase activities increased in response to silencing *SlSWEET7a* and *SlSWEET14*. In addition, cytoplasmic invertase and vacuolar invertase activities increased and could promote the hydrolysis of sucrose in the MG fruits of the SlSWEET7ai and SlSWEET14i lines. The higher amounts of hexose that accumulated in the SlSWEET7ai and SlSWEET14i lines may be attributed to the elevated invertase activities. Interestingly, sucrose concentrations increased in the MG fruits of the SlSWEET7ai and SlSWEET14i lines. There were no drastic differences in the expression of *SlSUT* genes between the wild-type and transgenic fruits, but the transcript levels of *SlHT1* and *SlHT2* decreased in the SlSWEET7ai lines. SWEETs may facilitate sucrose or hexose transport across membranes, as previous reports have also shown functional redundancy among SWEET family members^45^. Thus, in addition to SlHTs and SlSUTs, some SWEETs may facilitate hexose and sucrose transport. Among the *SlSWEETs* investigated, the expression of *7b*, *11* (*11b* or *11c*), *12c*, and *16* was upregulated in both RNAi lines. VvSWEET7 is a mono- and disaccharide transporter in grape^46^; moreover, AtSWEET16, a bidirectional vacuolar sugar facilitator, plays key roles in maintaining sugar homeostasis, and overexpression of *AtSWEET16* results in a decreased fructose contents in leaves^47^. Compounds *7b* and *16* could also play important roles in regulating sugar homeostasis. AtSWEET11 and AtSWEET12 from clade III were identified as key players in sucrose efflux from phloem parenchyma cells for phloem loading, and the sucrose content increased in the *atsweeet11;12* double mutant^20^. In the present study, suppression of *SlSWEET7a* and *SlSWEET14* resulted in a sucrose concentration decrease in mature leaves but an increase in MG fruit sucrose concentrations in the SlSWEET7ai and SlSWEET14i lines. This indicated that suppression of *SlSWEET7a* and *SlSWEET14* could promote increased sucrose allocation from the leaves to MG fruits; compounds *11* and *12c* may play key roles in this process. Accordingly, SlSWEET7a and SlSWEET14 may be important limiting factors regulating sucrose unloading in tomato fruits and may play an important role in maintaining cytosolic sugar homeostasis.

In addition, *FK* and *HK* are involved in hexose metabolism, hexose sensing, and signaling by phosphorylating hexose^48,49^. The transcript levels of *FK* and *HK* showed different patterns in the MG fruits of the SlSWEET7ai and SlSWEET14i lines. *SPS* overexpression increases sucrose turnover and the amount of sucrose unloaded in fruits^9,50^. Although two different SPS-encoded genes were induced by silencing *SlSWEET7a* and *SlSWEET14*, we nevertheless observed increased sucrose concentrations in the MG fruits of the SlSWEET7ai and SlSWEET14i lines. Furthermore, CWIN, encoded by the *LIN* gene, can influence the sugar composition in fruits, and the increase in CWIN activity by silencing its inhibitor was shown to increase fruit hexose levels^42^. *LIN5* was upregulated in the SlSWEET7ai line, and *LIN7*, *LIN8*, and *LIN9* were induced in the SlSWEET14 line. Cytoplasmic invertase (CIN), encoded by *CIN*, and vacuolar invertase (VI), encoded by *VI*, can regulate the sucrose/hexose ratio and lead to increased hexose levels and decreased cytosolic sucrose levels^2,51^. Moreover, *CIN1* was induced in the SlSWEET7ai line, and *VI* was significantly upregulated. These results showed that silencing of *SlSWEET7a* and *SlSWEET14* could influence sugar metabolism. Overall, *SlSWEET7a* and *SlSWEET14* are two possible key players in sucrose unloading and sugar metabolism in tomato fruits.

### *SlSWEET7a* and *SlSWEET14* could be regulated by phytohormones or sugars and could affect the growth and development of tomato

Sugars are not only important carbon and energy sources and structural constituents of cells but also essential signaling molecules^52^. The expression of various genes in plants is affected by sugars. The activity of SWEETs can also be feedback regulated by their substrates^21,53^. Our findings demonstrate that *SlSWEET7a* and *SlSWEET14* could respond to sugars (Supplementary Fig. 4). In addition, sugar signaling and the phytohormone pathway may exhibit crosstalk to modulate critical growth processes such as embryo establishment, seed germination, and seedling and tuber growth^49,54^. Notably, many sugar-responsive and hormone-responsive elements were found to be present in the promoters of *SlSWEET7a* and *SlSWEET14* (Supplementary Table S4, Supplementary Table S5). These phytohormones may thus have an important role in their regulation, as SlSWEET7a and SlSWEET14 could respond to phytohormones (Supplementary Fig. 4). These results show that *SlSWEET7a* and *SlSWEET14* might be involved in biological activities regulated by different phytohormones or sugars.

We found that the transgenic fruits with silenced *SlSWEET7a* were larger than the WT fruits. Silencing *SlSWEET7a* improved hexose accumulation. Moreover, altered sugar signaling may promote cell expansion and generate larger fruits. In addition, vacuolar invertase (VI) could promote cell expansion through crosstalk between sugar signaling and cell wall-associated kinases or phytohormones^1^. Therefore, the larger size of fruits of the *SlSWEET7a*-silenced lines may also be attributed to the elevated expression of *VI*. Similar results have been obtained when the apple hexose transporter MdHT2.2 is overexpressed—increased hexose concentrations and fruit sizes^55^. SlSWEET7a and SlSWEET14 probably have multiple physiological functions depending on their tissue localization. As reported in previous studies, overexpression of *SlSWEET1a* in tomato tissues decreased the hexose content in the fruits, virus-induced gene silencing of *SlSWEET1a* reduced hexose accumulation in young leaves^7,30^, and loss of *SlSWEET15* function inhibited fruit and seed development^31^. It has also been reported that plant height can be affected by altered expression of *SWEET* genes. For example, rice plants were shorter than WT rice plants when *OsSWEET14* was overexpressed^56^. In the present study, we also observed significantly increased height of the silenced lines. *SlSWEET7a* and *SlSWEET14* may also regulate plant growth and development via crosstalk among signaling pathways. As reported previously, FT-like protein (StSP6A) interacts with StSWEET11 to mediate source-sink regulation and affect tuber development via photoperiodic and sucrose signaling pathways^24^. However, the mechanism by which *SlSWEET7a* and *SlSWEET14* regulate the growth and development of tomato needs further clarification.

In the present study, we identified two *SlSWEET* genes, namely, *SlSWEET7a* and *SlSWEET14*, that are capable of transporting hexose and sucrose molecules across the plasma membrane. These two genes, which are mainly expressed in the fruits, could be directly or indirectly involved in sucrose unloading in tomato fruits. In addition, silencing *SlSWEET7a* and *SlSWEET14* affected hexose accumulation in tomato fruits and influenced vegetative and reproductive growth.

## Materials and methods

### Plant material and growth conditions

Tomato (*Solanum lycopersicum* L. ‘Micro-Tom’) seeds were used for genetic transformation in our study. Both wild-type tomato plants and transgenic tomato plants were grown in a greenhouse with a 16 h/8 h (light/dark) photoperiod at 25 °C and a relative humidity of 50–70%. Anthesis dates were determined according to the number of days after the sepals were removed. Wild-type tomato fruits were collected 2 days before anthesis and 0, 2, 4, 7, 14, and 21 days after anthesis and at the mature green (MG), breaking color (BC), and red ripe (RR) stages for expression analysis. In addition, different tissues from at least three plants, including the peduncle, sepal, total pericarp, placenta, vascular bundle, and septum tissues, were sampled at the MG, BC, and RR stages for expression analysis. Transgenic tomato fruits and leaves from at least five plants were collected at the mature green (MG) and red ripe (RR) stages to measure sugar and starch contents, and at least two fruits of each plant at each stage were sampled and pooled together. At each sampling point, at least three fruits (except for the transgenic lines) or three mature leaves (from the same position on each plant) from at least three plants were randomly selected and pooled together, and samples from each plant constitute one biological replicate. All the samples were immediately frozen in liquid nitrogen and kept at -80 °C until analysis.

### RNA isolation and quantitative real-time PCR

Total RNA was extracted from the different samples using a Simple Total RNA Kit (Tiangen, Beijing, China). cDNA was synthesized for use in quantitative real-time PCR assays as described previously^32^. Data analysis was carried out using Bio-Rad CFX Manager Software, following the 2^-∆∆CT^ method. The tomato housekeeping gene *ACTIN* was used as an internal control. All the primers used are listed in Supplementary Table S1 and Supplementary Table S2.

### Histochemical GUS activity assays

Promoters for *SlSWEET7a* (−1486 bp) and *SlSWEET14* (−1579 bp) were introduced into a pBGWES7.0 vector (including a GUS gene-coding region). The resulting fusion vector was transformed into Micro-Tom. GUS activity was examined using a GUS histochemical assay kit (Real-Time, Beijing, China) according to the manufacturer’s protocol, and the tissues in which GUS was expressed were observed and imaged under a Nikon SMZ800 stereomicroscope.

### Generation of RNAi plants

*SlSWEET7a* (*Solyc08g082770*) and *SlSWEET14* (*Solyc03g097560*) gene fragments of 120 and 144 bp, respectively, were amplified from tomato fruit cDNA using gene-specific primer pairs. The resulting product was cloned into a TOPO Gateway entry vector (Invitrogen, Gibco, America). The cloned fragment was subsequently transferred into a pB7GWIWG2(I) destination vector using LR Clonase II enzymes (Invitrogen, Gibco, America) according to the manufacturer’s instructions. The above constructs were individually introduced into *Agrobacterium tumefaciens* strain GV3101. Tomato transformation was subsequently performed according to a previously described method^57^. The presence of transgenes in PPT-resistant plants was confirmed by PCR using a vector-specific Bar gene primer (Supplementary Table S3). Seeds obtained from the transformants were grown in growth media consisting of three parts peat, one part perlite and one part vermiculite (v/v/v) in a glasshouse to obtain plants of the next generation (T_1_). The T_1_ generation plants were subjected to screening by spraying herbicides at a concentration of 60 μg/ml. The transgenic lines were subsequently grown to obtain nonsegregating homozygous lines. Out of 7 and 15 independent transgenic tomato lines for *SlSWEET7a* and *SlSWEET14*, respectively, three *SlSWEET7a* and two *SlSWEET14* lines of the T_4_ generation were selected and used for further analysis.

### Split-ubiquitin yeast two-hybrid assays

The coding regions (without the ATG codon and the stop codon) of *SlSWEET7a* and *SlSWEET14* with *SfiI* were amplified and cloned into a pBT3-STE bait vector, and the coding regions of SlSWEET7a and SlSWEET14 with *SfiI* were amplified and cloned into a pPR3-N prey vector. Then, the resulting vectors were transformed into yeast strain NMY51 according to the user manual (Dualsystems Biotech). Yeast transformation was performed with PEG/LiAc using a Yeastmaker Yeast Transformation System 2 Kit (Clontech, Takara, Japan). The yeast transformants were plated onto selection media lacking leucine and tryptophan but supplemented with β-galactosidase to confirm positive clones. The yeast transformants were also spread onto SD media lacking histidine, leucine, tryptophan, and adenine and supplemented with 10 mM 3-amino-1,2,3-triazole (3-AT), which was required to prevent autoactivation of the bait vector. All the assays were repeated independently at least three times, each yielding comparable results.

### Bimolecular fluorescence complementation assays

The coding sequences of the two SWEETs (without stop codons) were cloned into a Gateway pDONR221 entry vector and pXNGW (YFP) and pXCGW (CFP) Gateway destination vectors^58^. All the vectors were transformed into *Agrobacterium tumefaciens* strain GV3101. The fusion proteins were subsequently cotransformed into *N. benthamiana* leaves. Three days after transformation, the fluorescence signals were imaged using a confocal laser scanning microscope (Leica SP8, Germany). The fluorescence signals were observed at an excitation wavelength of 488 nm and emission wavelengths of 500-572 nm. Each BiFC assay was repeated three times, and consistent results were obtained.

### Subcellular localization of SlSWEET7a and SlSWEET14

The coding regions of the two *SWEET* genes (without stop codons) with *XbaI* and *KpnI* cleavage sites were cloned into a pCAM35-GFP expression vector. The resulting constructs were then transformed into *A. tumefaciens* strain GV3101 and used to infect *N. benthamiana* epidermal cells. mCherry-labeled AtPIP2A was used as a PM marker^59^. The experiments were performed independently three times. GFP fluorescence signals were observed with a confocal laser scanning microscope (Leica SP8, Germany). The fluorescence signal was observed at excitation wavelengths of 488 nm or 561 nm and emission wavelengths of 500–572 nm or 605–635 nm.

### Complementation assays for SlSWEET7a and SlSWEET14 in yeast

For complementation assays in yeast (*Saccharomyces cerevisiae*) cells, the ORFs of the two *SWEETs* with *XhoI* and *BamHI* were cloned into the yeast expression vector pDR195^16^. Subsequently, the resulting constructs were transformed into the hexose transport-deficient yeast strain EBY.VW4000 and the sucrose uptake-deficient yeast strain SUSY7/ura3. Transformants of the hexose transport-deficient strain were grown on liquid SD (synthetic deficient)/-uracil media supplemented with 2% maltose (glucose was used for the sucrose uptake-deficient strain). Serial dilutions of yeast cell suspensions of EBY.VW4000 were added dropwise onto solid SD/-uracil media consisting of either 2% maltose or 2% glucose/fructose. Similarly, serial dilutions of yeast cell suspensions of SUSY7/ura3 were added dropwise onto solid SD/-uracil media consisting of 2% glucose or 2% sucrose. Growth was documented via imaging after 3–4 days of growth at 30 °C.

### Enzyme assays and measurements of soluble sugars and starch

Fruits (0.5 g) at the MG stage were used for invertase activity analysis, as described previously^51^. Sucrose, glucose, and fructose were extracted in 80% (v/v) ethanol and analyzed via high-performance liquid chromatography (HPLC), as described previously^60^. Starch was extracted in 9.2 mol/L perchloric acid from the insoluble residue after soluble sugars were extracted and then filtered using filter paper^29^. The supernatant and distilled water were then mixed. The starch content was determined by the anthrone colorimetric method at 625 nm and calculated according to a standard curve of glucose samples. Fruits (0.5 g) at the MG and RR stages and mature leaves (0.2 g) from each of the different silenced lines were analyzed for their sugar concentration as well as starch level. The soluble solid content was determined using a hand-held sugar measurement instrument (PAL-fu, ATAGO, Japan); the fruit juice of each fruit was used to obtain a reading.

### Statistical analysis

The data are presented as the means ± SDs (standard deviations). Significance tests were carried out using SPSS software (version 17.0) based on Student’s *t* tests at *P* < 0.01 or *P* < 0.05.

## Supplementary information

Supplemental material Rivised
