# Supplementary material for: Plasma membrane-localized SlSWEET7a and SlSWEET14 regulate sugar transport and storage in tomato fruits
Source: Hortic Res. 2021 Aug 1;8:186. doi: 10.1038/s41438-021-00624-w (PMC8325691; doi:10.1038/s41438-021-00624-w)
Supplement: Supplementary file 1 — Supplemental material Rivised [file 41438_2021_624_MOESM1_ESM.doc]

Supplementary Material

Supplementary Table S1. The primers used for qRT-PCR analysis of *SWEETs*

| Genomic ID | Gene name | Sense primer sequence (5'-3') | Adverse primer sequence (5'-3') |
| --- | --- | --- | --- |
| Solyc04g064610 | *SlSWEET1a* | TTTGCTGCTGTAGCCCTTGTT | GAACTCCACGCTCTTCGTCTTG |
| Solyc04g064620 | *SlSWEET1b* | TGCCTTTCTTCTTGTCCTTG | TGGGTGTTAGATGGCTTCTCT |
| Solyc04g064630 | *SlSWEET1c* | TTCCATAGGTGGTGCCTT | CTTTCCCAAGCAAGCCAT |
| Solyc04g064640 | *SlSWEET1d* | ATTGCTTGCTCTCAGTTTGG | ACAACGAGGTATCCGCAGA |
| Solyc06g060590 | *SlSWEET1e* | GGATTGTTGGAAATGCCGC | GCAGAAAGCAAGCAGTTGAG |
| Solyc06g060580 | *SlSWEET1f* | CATTTGCCGCTATCCTATCTTG | TTCCTAAAGTCAACCCAACACC |
| Solyc02g071520 | *SlSWEET2a* | TATGCCTCTGGTATGGGACACC | TATCCAACGAAAGTTTGCCGAC |
| Solyc07g062120 | *SlSWEET2b* | ATCTGGAACTCCGACGCAT | AGGCACTCATTAGGAAGGTGG |
| Solyc03g007360 | *SlSWEET3* | TCCAAATCTGGTGGGAAC | TCAGTGAGAACGAGCATCG |
| Solyc03g114200 | *SlSWEET5a* | AATGGAGGTATTTGGTTCGCCT | TTTCTTCATCGTCCCAGTTGG |
| Solyc06g071400 | *SlSWEET5b* | TTGCTGTTTGGATGCGTG | CAATGATTCCTACAACCGTCC |
| Solyc02g086920 | *SlSWEET6a* | TCGTTCATCCACACAGCATTC | ATTCCAAAGGCGGTAGCG |
| Solyc08g082770 | *SlSWEET7a* | TGATGCCTACATTCTCGCACC | TCCTTTAGCCTCTCTTGCTGCC |
| Solyc12g055870 | *SlSWEET7b* | TGCCCTTCTTCCTTTCCCT | CATCCTACCAAACCCACTCC |
| Solyc03g097580 | *SlSWEET10a* | GAGTGAGAGACAAAGTGCTAGAAA | AGCCCAATGACCAGAAATACC |
| Solyc03g097600 | *SlSWEET10b* | ACATTGTGAGGCTTGGTTTAATG | TGCAACTTGGGCTCATTCT |
| Solyc03g097610 | *SlSWEET10c* | GATGGCTATTGCTGGTCATTGG | ACCCTGGCTTTCTTTGGTGC |
| Solyc03g097870 | *SlSWEET11a* | GAG ATA CAA AAG ACT GAA G | TGG CTA CGT TTG TTT TAT CAG TAA C |
| Solyc03g097570 | *SlSWEET11b* | CAGGACTCACAGAGGAACAAA | CATTGCGTCGTGCAAACA |
| Solyc06g072620 | *SlSWEET11c* | ATGGAAATTTAACTGAGGTTGCT | AACATGCTTGCAAATTTACAAATTA |
| Solyc06g072640 | *SlSWEET11d* | TCTAGGCGGATTGGTTGAGG | ACCTTGGCTTTCTTTGGTGC |
| Solyc03g097590 | *SlSWEET12a* | TGGTTTACTCGTTTGCTCAGATA | TTTGCAGCTTAGGCATGTTTAC |
| Solyc03g097620 | *SlSWEET12b* | GTCTATGTCGCGGTACCAAATA | TGCTCTTGATCCACCTTTACC |
| Solyc05g024260 | *SlSWEET12c* | GCATCGTGTTTCAAGTGGTTCG | TCTATCGCTGGCTTTGCGTT |
| Solyc06g072630 | *SlSWEET12d* | TGGTTCACTGATTTCATGTACA | TATTTCGAGTGTTTTCCGCAGC |
| Solyc03g097560 | *SlSWEET14* | TGAGTGCAATTGTGTGTTCAGA | GGCCTCTATGATTGCCTTTGG |
| Solyc09g074530 | *SlSWEET15* | CATTGCGATGCCAAACATTC | TATCTCCACCACCACCGCTT |
| Solyc01g099880 | *SlSWEET16* | CTTATGTTCGCTGCTCC | ACTACCATTGACTGTGACCACT |
| Solyc01g099870 | *SlSWEET17* | TGGTGGCGTTTGGACATTGT | GGCAGCAAGTGTTCCGTTTG |

Supplementary Table S2. The primers used for qRT-PCR analysis of sucrose metabolism genes and sucrose transporter genes

| Gene name | Sense primer sequence (5'-3') | Adverse primer sequence (5'-3') |
| --- | --- | --- |
| *FK1* | GTTGCAGCCAACGAGACATG | AAGGCTTTCCAGCATTTCCTT |
| *FK2* | CGACGATGAGTTCGGTCACA | GGCGCCCTTGTCAAAATTAA |
| *FK3* | AGATGCGCTGTCAAAGCAACT | CACTTGGCAATGCTCTTCCTT |
| *HK1* | TCTTGCTGCCTCTCATTCAATG | TTCCCTCTAGCAAGATATGGCTTT |
| *HK2* | TTCCTGGGAATGCCAACATATT | GGGAGAATTAGCAGTTTGCTGAGT |
| *HK3* | CTTGCTAAAGGGAACCCACTTCT | GCATGACACAGTGCACAAGGA |
| *HK4* | ACAGAGCTACTAGGCTCGGAAA | GCAGCTAATAATGCAGCTCCAA |
| *LIN5* | CTGAATGCTTGGAGCATGGAT | GAGGATTTTTGTGAACATCATCTACTG |
| *LIN6* | AGCACATTTATTCGCCTTCAACAA | CTTTGTGACGTGGCATAATAAGAT |
| *LIN7* | GAACGGAGCCAATCACAATTG | TCCCCCTTTTACCATAGTTCCTT |
| *LIN8* | AGTCATTGAATGCATGGAGCAT | GAGCAATCAAATACGTCACCACAA |
| *LIN9* | ACTGGGTCAACCAACGAATC | TGCCCTCATACTTGATCCAT |
| *SPSA2* | GGAAGTTGCAGCAAGGAAGATATT | TTTAACCTCCTCAGCGGAAATC |
| *SPSB* | CCTGTGGCACGAGTGTAGGA | TTCCATTGCGGGTGTCTCAT |
| *VI* | TCCTTCCCTTTGCAAGACTTGT | TCTCCCTCTTCCCTTTCTTGATG |
| *SS1* | CAAGTACCGTAAGATGGCTGAA | TGAAACACTAAACAGAAGCCTCTAC |
| *SS3* | TTTAGAAATTGTTTGCCCCACAT | CTACAGAAGGGAAAAATGGCAAAT |
| *SS4* | AGTTCCGTGAGTTGGTAAAATCTGT | TGGTATGCTCCACGTTATTTATTCC |
| *CIN1* | TGCCATCAACAACCGTCT | TCCAATCCACAAGCCAAG |
| *CIN2* | TGGTCCATTGTGTCGTCTT | GGTGTGTTCTTAGGGTCGCT |
| *CIN3* | CGTAGAATGGGTGTGTATGG | GAACTTGTTGACTGCTGTGTG |
| *CIN4* | AGATGTGCCTTGTTTCTCCT | CCGATGAAGTAACCACCAC |
| *CIN5* | TCTTGTCTTCCCTTGCCA | GGTGCTTCCTTGCTTGTTTAC |
| *CIN6* | TTGTTACGTGCGTATGGGAA | CCTGAATCTCCAAAGGGTGA |
| *CIN7* | CGTGGTGGTTACTTTGTTGG | TCCCAGCGTGCTTCTATGA |
| *CIN8* | TGGGAAATGCTCTGGAGA | GTCACCAACAAAGTCGGAA |
| *HT1* | CGATGATCGAACGTGGTAAC | CAACAAGTTCCTCCAGGGAT |
| *HT2* | TCAACTACGGAACAGCCAAG | TCAGGTTCAATGTTGTCGGT |
| *HT3* | TGCGATAGCACAGTCTTTCC | CTTCGTTTCAGGCAAGAACA |
| *SUT1* | TTCCATAGCTGCTGGTGTTC | TACCAGAAATGGGTCCACAA |
| *SUT2* | CCTACAGCGTCCCTTTCTCT | GGATACAACCATCTGAGGTACAA |
| *SUT4* | TCTCCGCTGATATTGGATGG | GCAACATCGAGAAGCCAAAA |
| *ACTIN* | TGTCCCTATTTACGAGGGTTATGC | AGTTAAATCACGACCAGCAAGAT |

Supplementary Table S3. The primers used for generating expressing constructs and identification

| Primer name | primer sequence (5'-3') | Purpose |
| --- | --- | --- |
| AtSWEET1-F | CCTCGAGAGAATCTTTAGCCACCTT | Yeast complementation assays |
| AtSWEET1-R | CGGATCCGCAGAGTTAATACGAAATCACA |
| SlSWEET7a-F | CCTCGAGACATAGCTATGACTTTTAATAG |
| SlSWEET7a-R | CGGATCCTGAAAAACAGAAGGCCCAAT |
| SlSWEET14-F | CCTCGAGCTATG ACGACTCATT TGGCTTT |
| SlSWEET14-R | CGGATCCCACACACAATCAGACCTATG |
| GWSlSWEET7a-F | GGGGACAAGTTTGTACAAAAAAGCAGGCTTCGGTACCATGACTTTTAATAGGGACAA | Y2H assays or BiFC assays |
| GWSlSWEET7a-R | GGGGACCACTTTGTACAAGAAAGCTGGGTGACTAGTAACTCTAAAATCACTTCTCG |
| GWSlSWEET14-F | GGGGACAAGTTTGTACAAAAAAGCAGGCTTCGGTACCATGACGACTCATTTGGCTTT |
| GWSlSWEET14-R | GGGGACCACTTTGTACAAGAAAGCTGGGTGACTAGTAGAGGCCTCTATGATTGCCT |
| SlSWEET7ai-F | CACCTGATGCCTACATTCTCGCACC | RNAi silencing |
| SlSWEET7ai-R | TCCTTTAGCCTCTCTTGCTGCC |
| SlSWEET14iF | CACCGCCAAAGGCAATCATAGAGG |
| SlSWEET14iR | AGGCACACACAATCAGACCT |
| G7aF | GGTACCATGACTTTTAATAGGGACAATGCT | Subcellular localization assays |
| G7aR | TCTAGAAACTCTAAAATCACTTCTCGGGTTT |
| G14F | GGTACCATGACGACTCATTTGGCTTTTGTAT |
| G14R | TCTAGAAGAGGCCTCTATGATTGCCTTTGGC |
| GUS7aF | CACCATTTAAGTAAAAAATAAAACACAGAAGTG | Tissue-specific localization assays |
| GUS7aR | TGAATCAAAGAATGAAGATGAGAATAGCC |
| GUS14F | ACTCAGTCCTTGCTTATCTGTTCG |
| GUS14R | AATACAAAAGCCAAATGAG |
| Bar-F | GAAGTCCAGCTGCCAGAAA- | Identification |
| Bar-R | GAAGTCCAGCTGCCAGAAA | Identification |

Supplementary Table S4. Prediction of cis-acting elements of *SWEET7a* promoter

| *Cis*-acting element | Sequence | Response | Number  of copies |
| --- | --- | --- | --- |
| WRKY71OS | TGAC | Pathogen; gibberellin | 17 |
| WBOXNTERF3 | TGACY | Wounding | 13 |
| WBOXHVISO1 | TGACT | Sugar | 9 |
| WBOXATNPR1 | TTGAC | Stress; SA | 13 |
| TBOXATGAPB | ACTTTG | Light | 4 |
| TATCCAYMOTIFOSRAMY3D | TATCCAY | Sugar repression | 3 |
| TATCCAOSAMY | TATCCA | Sugar and hormone regulation | 3 |
| T/GBOXATPIN2 | AACGTG | Jasmonate (JA) | 1 |
| TCCC-motif | TCTCCCT | Light | 1 |
| TGACG-motif | TGACG | MeJA | 1 |
| SURECOREATSULTR11 | GAGAC | Sulfur | 1 |
| SURE2STPAT21 | AATACTAAT | Sucrose | 1 |
| SREATMSD | TTATCC | Sugar repression | 3 |
| SORLIP5AT | GAGTGAG | Light | 1 |
| PYRIMIDINEBOXOSRAMY1A | CCTTTT | Gibberellin; Sugar repression | 1 |
| PREATPRODH | ACTCAT | Pro- or hypoosmolarity | 3 |
| NTBBF1ARROLB | ACTTTA | Auxin | 1 |
| MYCCONSENSUSAT | CANNTG | Dehydration; cold; ABA | 10 |
| MYCATRD22 | CACATG | Dehydration; ABA | 1 |
| MYCATERD1 | CATGTG | Dehydration | 1 |
| MYBGAHV | TAACAAA | Gibberellin; Sugar repression | 1 |
| MYBCORE | CNGTTR | Water stress; dehydration | 2 |
| MYBATRD22 | CTAACCA | Dehydration; ABA | 1 |
| MYB2CONSENSUSAT | YAACKG | Dehydration; ABA | 1 |
| MYB1AT | WAACCA | Dehydration; ABA | 3 |
| MBS | CAACTG | Drought | 1 |
| MRE | AACCTAA | Light | 1 |
| LTR | CCGAAA | Low temperature | 2 |
| INRNTPSADB | YTCANTYY | Light | 11 |
| IBOXCORE | GATAA | Light | 8 |
| IBOX | GATAAG | Light | 1 |
| GT1GMSCAM4 | GAAAAA | Pathogen; salt | 14 |
| GT1-motif | GGTTAA | Light | 1 |
| GT1CONSENSUS | GRWAAW | Light | 37 |
| GATABOX | GATA | Light | 26 |
| GAREAT | TAACAAR | Gibberellin | 2 |
| [G-Box](http://bioinformatics.psb.ugent.be/webtools/plantcare/cgi-bin/show_site_info.htpl?QWhere=ID_of_Site like 'CACGTT'&StartAt=0&NbRecs=10) | CACGTT | Light | 1 |
| GARE-motif | TCTGTTG | Gibberellin | 1 |
| [ERE](http://bioinformatics.psb.ugent.be/webtools/plantcare/cgi-bin/show_site_info.htpl?QWhere=ID_of_Site like 'ATTTTAAA'&StartAt=0&NbRecs=10) | ATTTCAAA | Ethylene | 3 |
| DPBFCOREDCDC3 | ACACNNG | ABA | 1 |
| CGTCA-motif | CGTCA | MeJA | 1 |
| CURECORECR | GTAC | Copper; oxygen | 2 |
| CPBCSPOR | TATTAG | Cytokinin | 2 |
| chs-CMA1a | TTACTTAA | Light | 1 |
| ARR1AT | NGATT | Cytokinin | 29 |
| ANAERO1CONSENSUS | AAACAAA | Anaerobic induction | 2 |
| AMYBOX2 | TATCCAT | Sugar starvation | 3 |
| ACGTATERD1 | ACGT | Dehydration; dark-induced senescence | 6 |
| ABRE | ACGTG | Dehydration; dark-induced senescence; ABA | 1 |
| [ARE](http://bioinformatics.psb.ugent.be/webtools/plantcare/cgi-bin/show_site_info.htpl?QWhere=ID_of_Site like 'AAACCA'&StartAt=0&NbRecs=10) | AAACCA | Anaerobic induction | 2 |
| AuxRR-core | GGTCCAT | Auxin | 1 |
| [Box 4](http://bioinformatics.psb.ugent.be/webtools/plantcare/cgi-bin/show_site_info.htpl?QWhere=ID_of_Site like 'ATTAAT'&StartAt=0&NbRecs=10) | ATTAAT | Light | 5 |
| -10PEHVPSBD | TATTCT | Light | 2 |

Supplementary Table S5. Prediction of cis-acting elements of *SWEET14* promoter

| *Cis*-acting element | Sequence | Response | Number  of copies |
| --- | --- | --- | --- |
| WRKY71OS | TGAC | Pathogen; gibberellin | 20 |
| WBOXNTERF3 | TGACY | Wounding | 10 |
| WBOXHVISO1 | TGACT | Sugar | 7 |
| WBOXATNPR1 | TTGAC | Stress; SA | 12 |
| TBOXATGAPB | ACTTTG | Light | 2 |
| TATCCAYMOTIFOSRAMY3D | TATCCAY | Sugar repression | 1 |
| TATCCAOSAMY | TATCCA | Sugar and hormone regulation | 1 |
| TATC-box | TATCCCA | Gibberellin | 1 |
| TC-rich repeats | ATTCTCTAAC | Defense and stress | 3 |
| TGACG-motif | TGACG | MeJA | 1 |
| SURECOREATSULTR11 | GAGAC | Sulfur | 4 |
| SURE1STPAT21 | AATAGAAAA | Sucrose | 1 |
| SEBFCONSSTPR10A | YTGTCWC | Pathogen | 3 |
| PYRIMIDINEBOXOSRAMY1A | CCTTTT | Gibberellin; sugar repression | 3 |
| PYRIMIDINEBOXHVEPB1 | TTTTTTCC | Gibberellin (GA) | 1 |
| PROXBBNNAPA | CAAACACC | ABA | 1 |
| P1BS | GNATATNC | Phosphate starvation | 4 |
| NTBBF1ARROLB | ACTTTA | Auxin | 5 |
| NRRBNEXTA | TAGTGGAT | Wounding and tensile stress | 2 |
| MYCCONSENSUSAT | CANNTG | Dehydration; cold; ABA | 14 |
| MYCATRD22 | CACATG | Dehydration; ABA | 1 |
| MYCATERD1 | CATGTG | Dehydration | 1 |
| MYBGAHV | TAACAAA | Gibberellin (GA) | 1 |
| MYBCORE | CNGTTR | Water stress; dehydration | 3 |
| MYBCORE | CNGTTR | Water stress; dehydration | 3 |
| MYB2CONSENSUSAT | YAACKG | Dehydration; ABA | 3 |
| MYB2AT | TAACTG | Dehydration | 1 |
| MYB1AT | WAACCA | Dehydration; ABA | 4 |
| INRNTPSADB | YTCANTYY | Light | 11 |
| IBOXCORE | GATAA | Light | 11 |
| IBOX | GATAAG | Light | 2 |
| HDZIP2ATATHB2 | TAATMATTA | Light | 1 |
| GT1GMSCAM4 | GAAAAA | Pathogen; salt | 9 |
| GT1-motif | GGTTAA | Light | 3 |
| GT1CONSENSUS | GRWAAW | Light | 37 |
| G-box | CACGTC | Light | 1 |
| GATA-motif | AAGATAAGATT | Light | 1 |
| GATABOX | GATA | Light | 26 |
| GAREAT | TAACAAR | Gibberellin (GA) | 2 |
| GADOWNAT | ACGTGTC | Gibberellin (GA) | 1 |
| ERE | AWTTCAAA | Ethylene | 4 |
| CURECORECR | GTAC | Copper | 6 |
| CPBCSPOR | TATTAG | Cytokinin | 3 |
| CBFHV | RYCGAC | Dehydration | 1 |
| CAREOSREP1 | CAACTC | Gibberellin (GA) | 1 |
| CGTCA-motif | CGTCA | MeJA | 1 |
| BIHD1OS | TGTCA | Disease resistance | 9 |
| [Box 4](http://bioinformatics.psb.ugent.be/webtools/plantcare/cgi-bin/show_site_info.htpl?QWhere=ID_of_Site like 'ATTAAT'&StartAt=0&NbRecs=10) | ATTAAT | Light | 8 |
| ARR1AT | NGATT | Cytokinin | 24 |
| ARFAT | TGTCTC | Auxin | 3 |
| ANAERO2CONSENSUS | AGCAGC | Anaerobic induction | 5 |
| AMYBOX2 | TATCCAT | Sugar starvation | 2 |
| AMMORESIIUDCRNIA1 | GGWAGGGT | Ammonium | 1 |
| ACGTATERD1 | ACGT | Dehydration; dark-induced senescence; | 4 |
| ACGTABREMOTIFA2OSEM | ACGTGKC | ABA | 1 |
| ACGTABOX | TACGTA | Sugar repression | 2 |
| [ABRE](http://bioinformatics.psb.ugent.be/webtools/plantcare/cgi-bin/show_site_info.htpl?QWhere=ID_of_Site like 'ACGTG'&StartAt=0&NbRecs=10) | ACGTG | ABA | 1 |
| ARE | AAACCA | Anaerobic induction | 2 |
| AT1-motif | AATTATTTTTTATT | Light | 1 |
| AuxRR-core | GGTCCAT | Auxin | 1 |
| -10PEHVPSBD | TATTCT | Light | 6 |


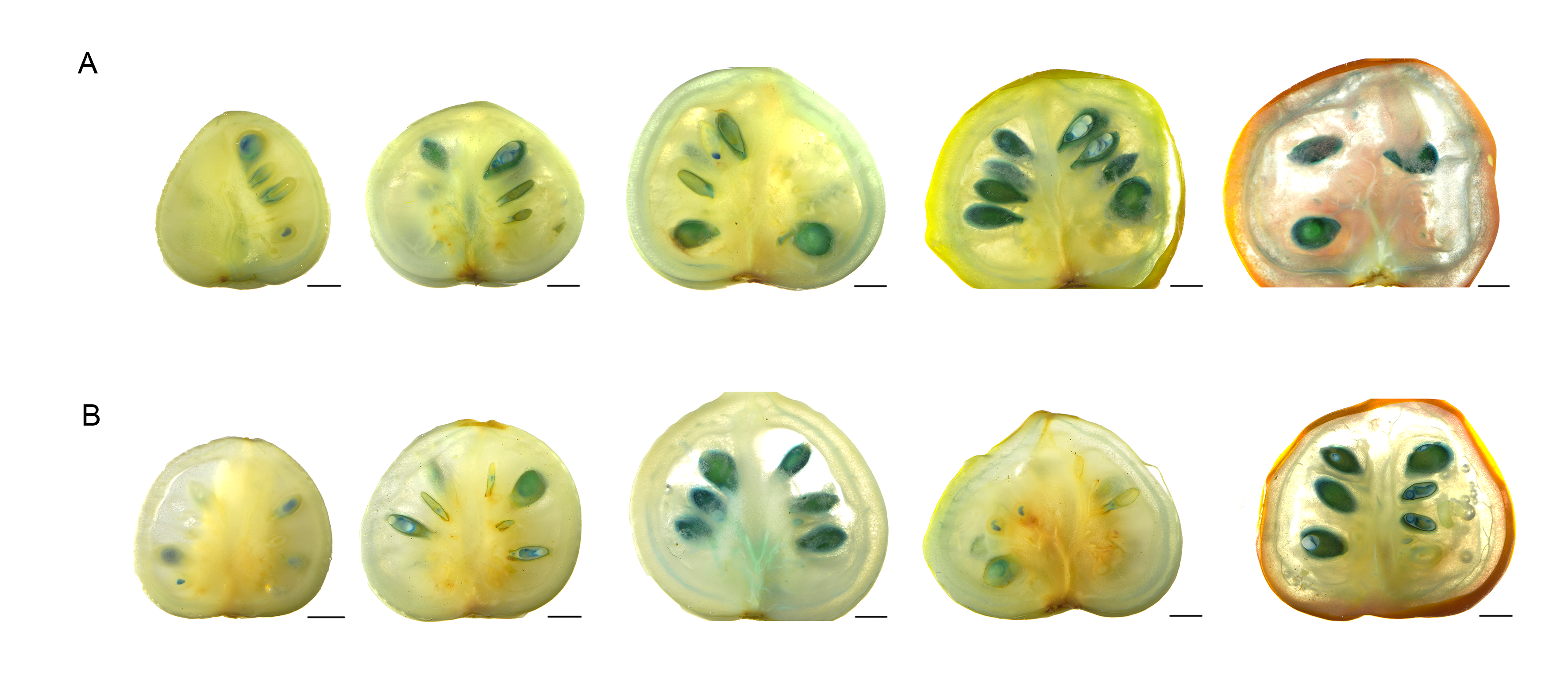


Supplementary Fig. 1 GUS staining of transient expression driven by *SlSWEET7*a and *SlSWEET14* promoter. Fruits on plants at different development stage were selected and injected buffer solution (20μmol AS, 6.25mol MES, 6.25mmol MgCl_2_·6H2O) containing agrobacterium transformed by the GUS vector of *SlSWEET7*a or *SlSWEET14* promoter. Treated fruits were harvested at 3 days later and used for GUS activities assays. (A-B) Histochemical staining of GUS activities driven by the promoter of *SlSWEET7*a and *SlSWEET14*, respectively. Scale bars are 2000μm.


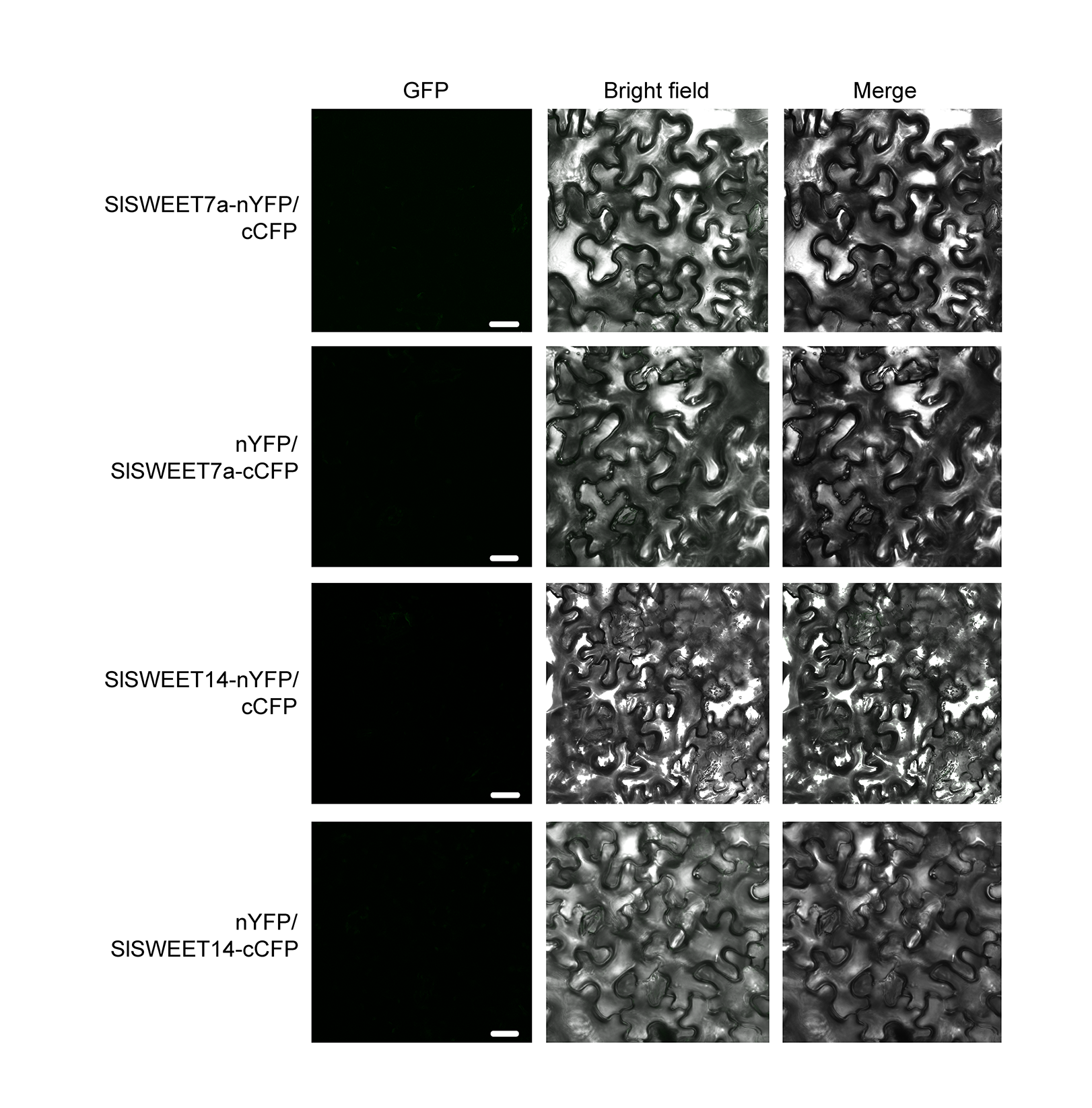


Supplementary Fig.2 Bimolecular fluorescence complementation assays for the negative controls for interaction of SlSWEET7a and SlSWEET14. Scale bars correspond to 25 μm


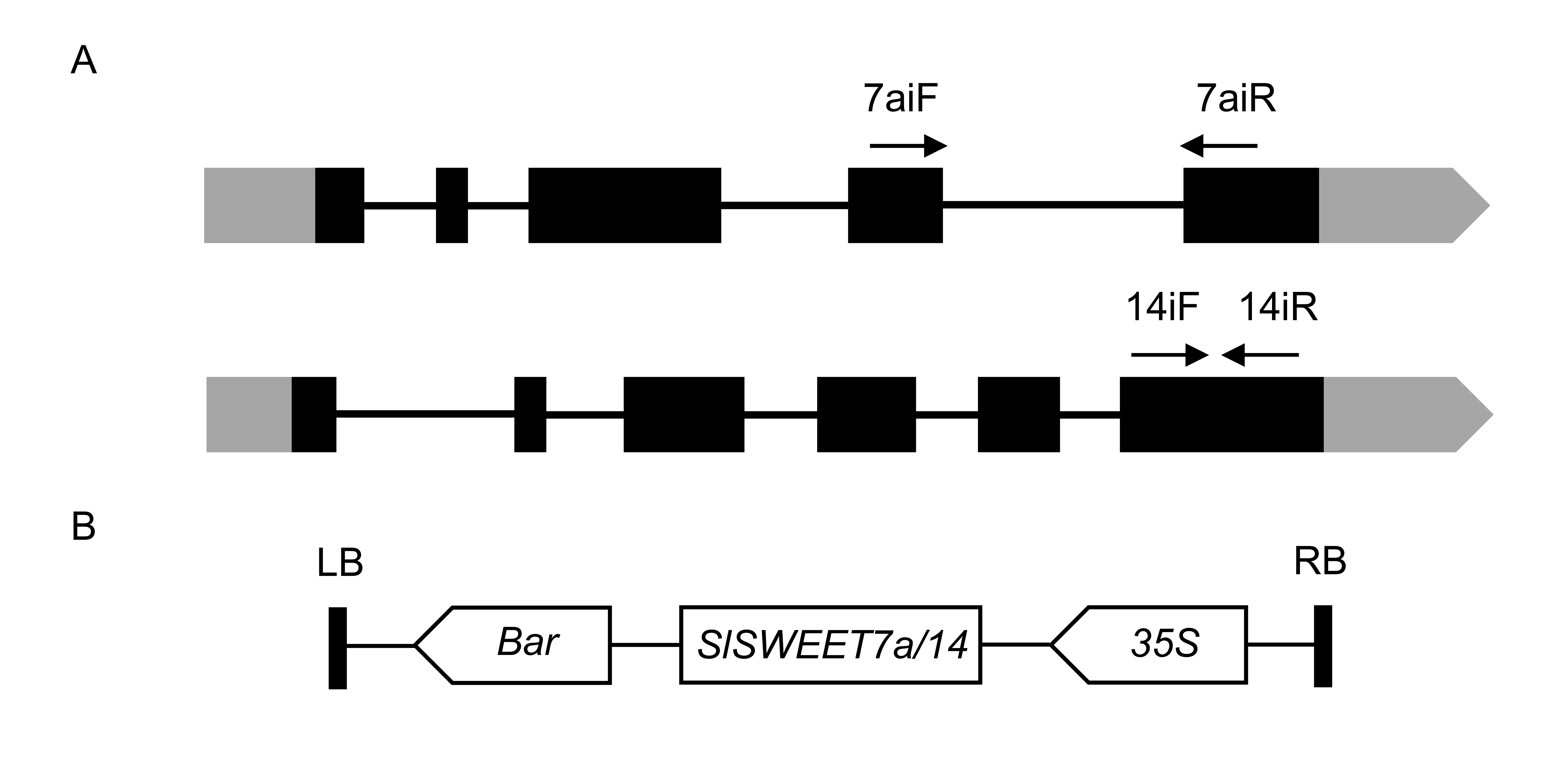


Supplementary Fig. 3 The structure of gene and resulting binary vector for RNAi interference. A, a schematic representation of the genomic structure of *SlSWEET7a/14* at top and bottom, respectively. The boxes filled by light and dark represent non-coding UTRs region and coding exons region, respectively. The solid lines show the introns region. Arrows indicate the position of primers used for RNAi construction. B, a schematic structure of resulting vector of *SlSWEET7a* or *14* used for RNAi.


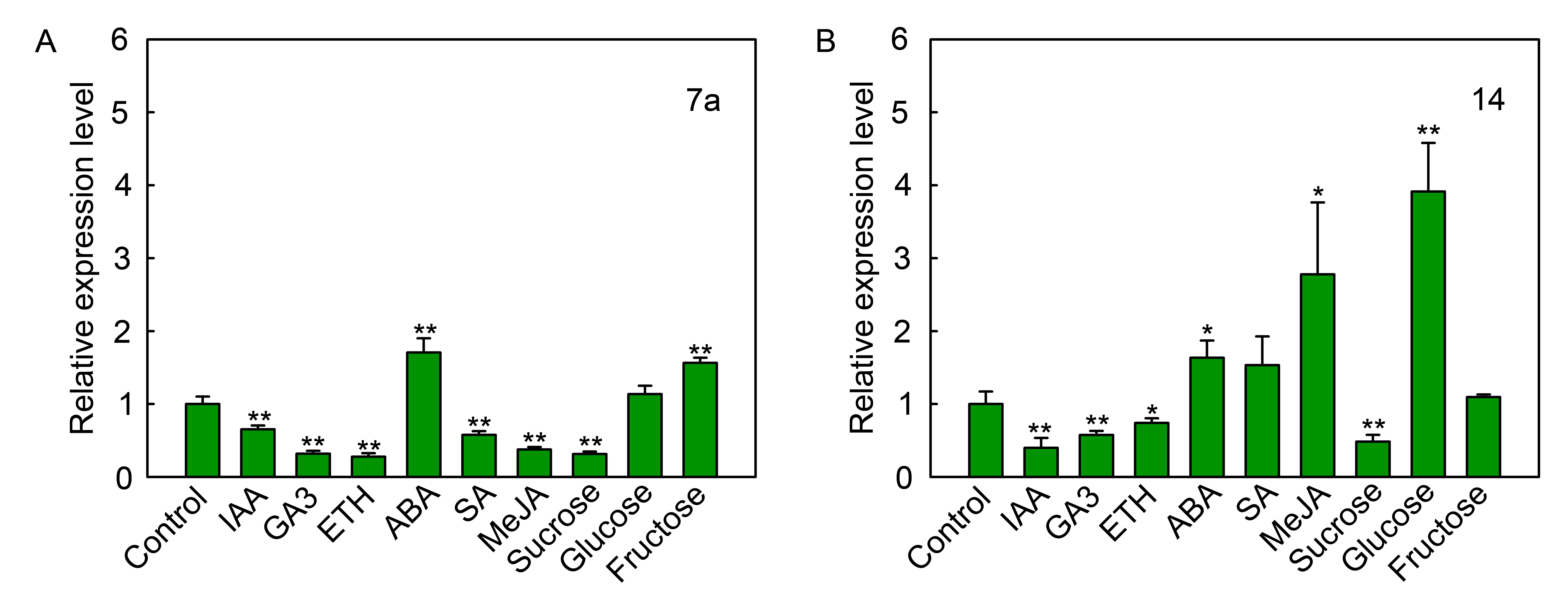


Supplementary Fig.4 The transcriptional level of SlSWEET7a and SlSWEET14 under different treatment on fruit discs.

The mixed cDNA of three SlSWEET7ai lines or two SlSWET14i lines was used for the experiments. (A, B) The expression level of SlSWEET7a and SlSWEET14 under different treatment at 24h latter, respectively. The expression data of control was normalized to 1. The ACTIN gene was used as internal control. Data represent mean values ± SD of three biological replicates. Asterisks indicate P values (*P <0.05, **P <0.01) according to Student’s t test.

Supplementary methods

Promoter Sequences Analysis of *SlSWEET7a* and *SlSWEET14*

An upstream sequence (2500 bp) above the start codon of *SlSWEET7a* or *SlSWEET14* was obtained from the SOL Genomics Network (http:// www.solgenomics.net) for better understand potential roles of them. The region of the promoter sequence of each gene was then analysed for the presence of putative *cis*-regulatory elements using the PlantCare database [(http://bioinformatic s.psb.ugent.be/webtools/plantcare/html/](../(http://bioinformatic%20s.psb.ugent.be/webtools/plantcare/html/)) and PLACE database (https://sogo.dna.affrc. go.jp/cgi-bin/sogo.cgi?lang=en&pj=640&action=page&page=newplace).

Incubation experiment of fruit discs

MG fruits collected at 35 days after flowering were used as samples. The incubation experiment was performed according to the methods previous described^1^. Fruit discs 6 mm in diameter and 1 mm in thickness were obtained using a hole puncher, divided into 14 groups (each group including four fruit discs) and performed for incubation assays as described in previous method^1^. One group was incubated in the buffer without any added sugar or hormones as control, the others were incubated in the buffer with 50 μmol/L of IAA, 100 μmol /L of GA_3_, 100 μmol/L of ethephon (ETH), 50 μmol/L of abscisic acid (ABA), 50 μmol/L of Me-JA, 100 μmol/L of SA, 100 mmol/L of sucrose, 100 mmol/L of fructose, and 100 mmol/L of glucose, respectively. The abovementioned hormones were all dissolved in 95 % (v/v) ethanol. The proportion of ethanol in each treatment was 0.475 % (v/v). All treatments were shaken at 25 ℃ for 24 h, after which the discs were immediately frozen in liquid nitrogen and stored at −80 °C until used. The experiment was performed independently three times.

References

1. Jia, *H*. et al. Abscisic acid and sucrose regulate tomato and strawberry fruit ripening through the abscisic acid-stress-ripening transcription factor*. Plant Biotechnol.* J**.** 14, 2045-65 (2016*).*
